# Imaging Unique DNA Sequences in Individual Cells Using a CRISPR-Cas9-Based, Split Luciferase Biosensor

**DOI:** 10.3389/fgeed.2022.867390

**Published:** 2022-03-25

**Authors:** Nicholas G. Heath, Henriette O’Geen, Nicole B. Halmai, Jacob E. Corn, David J. Segal

**Affiliations:** ^1^ Genome Center and Department of Biochemistry and Molecular Medicine, University of California, Davis, Davis, CA, United States; ^2^ Integrative Genetics and Genomics, University of California, Davis, Davis, CA, United States; ^3^ Innovative Genomics Institute, University of California, Berkeley, Berkeley, CA, United States; ^4^ Department of Biology, ETH, Zürich, Switzerland

**Keywords:** CRISPR, live cell imaging, split reporter, single gene locus, dCas9

## Abstract

An extensive arsenal of biosensing tools has been developed based on the clustered regularly interspaced short palindromic repeat (CRISPR) platform, including those that detect specific DNA sequences both *in vitro* and in live cells. To date, DNA imaging approaches have traditionally used full fluorescent reporter-based fusion probes. Such “always-on” probes differentiate poorly between bound and unbound probe and are unable to sensitively detect unique copies of a target sequence in individual cells. Herein we describe a DNA biosensor that provides a sensitive readout for such low-copy DNA sequences through proximity-mediated reassembly of two independently optimized fragments of NanoLuc luciferase (NLuc), a small, bright luminescent reporter. Applying this “turn-on” probe in live cells, we demonstrate an application not easily achieved by fluorescent reporter-based probes, detection of individual endogenous genomic loci using standard epifluorescence microscopy. This approach could enable detection of gene edits during *ex vivo* editing procedures and should be a useful platform for many other live cell DNA biosensing applications.

## Introduction

A critical bottleneck in the gene editing process is the ability to identify and isolate individual cells with desired edits within a population of treated cells. Current approaches typically require time-consuming and labor-intensive single cell isolation followed by isogenic population expansion (Mathupala and Sloan, 2009; Hu et al., 2016; Giuliano et al., 2019) and downstream *in vitro* analysis of DNA sequence (Bauer et al., 2015; Vouillot et al., 2015; Sentmanat et al., 2018; Ren et al., 2019) in a portion of the expanded population. However, cell types that exhibit low efficiencies of transfection, editing, single cell isolation, or population expansion can make these procedures particularly challenging (Bruenker, 2006; Tamm et al., 2016; Zhang et al., 2017a; Li et al., 2018; Modarai et al., 2018). Furthermore, homology directed repair (HDR) can exhibit extremely low efficiency in certain cell types (Liu et al., 2019).

A promising alternative strategy to validate gene edits could be the direct biosensing of user-defined sequences at single copy with single cell resolution. In recent years, the CRISPR/Cas gene editing system has been modified for imaging endogenous genomic loci, but the vast majority of current approaches utilize full-length fluorescent reporter-based probes, such as dCas9-GFP (Chen et al., 2013; Deng et al., 2015; Ma et al., 2015; Dreissig et al., 2017; Ye et al., 2017; Chen et al., 2018; Zhang et al., 2018; Wu et al., 2019). However, each full reporter sensor molecule produces a signal whether bound to its target DNA or not, resulting in a high fluorescent background that negatively impacts the signal-to-background ratio (SBR). For this reason, such “always-on” sensors must rely on obtaining a high local concentration of probes to distinguish signal from background, limiting their use to highly repetitive elements that can be targeted by one sgRNA or to unique sequences targeted by 20–30 or more sgRNAs (Chen et al., 2013; Chen et al., 2018). Imaging a short sequence present at a single copy is so far not possible.

However, “turn-on” DNA biosensors offer the possibility that signal could be produced only after binding of one or more subunits to a target sequence. Light production in such a system can occur either by activation of a chromophore by energy transfer from another activated chromophore or by reassembly of a bright reporter. However, recent efforts to apply Förster Resonance Energy Transfer (FRET) to sense DNA (Didenko, 2001; Boutorine et al., 2013; Dahan et al., 2013; Wu et al., 2018; Mao et al., 2019) have still required more than three unique sgRNAs, while split reporter DNA or RNA biosensing has been described mainly by previous studies *in vitro* (Stains et al., 2005; Ghosh et al., 2006; Ooi et al., 2006; Zhang et al., 2017b; Zhou et al., 2021) with several studies describing using transcription activator-like effectors (TALEs) as DNA binding domains and split fluorescent proteins for DNA biosensing in live cells (Hu et al., 2017; Hu et al., 2019).

In addition to higher background from unbound probes, fluorescence-based biosensing is plagued by issues with phototoxicity and photobleaching as well as a naturally high cellular auto-fluorescent background (Choy et al., 2003; Bernas et al., 2005; Tung et al., 2016), all of which negatively impact the SBR. To counteract these negative effects on biosensing SBR and increase sensitivity for an underlying physicochemical target, luminescent reporters could offer an attractive alternative in biosensing experiments. Cellular luminescent background signal is essentially nonexistent due to the fact that light is produced from a catalytic reaction of an enzyme with its substrate instead of from excitation by incident exogenous light (Tung et al., 2016). While luminescent reporters have the advantage of lower background, one historical advantage of fluorescent reporters is that they have remained brighter than available luminescent reporters (Hall et al., 2012). However, a relatively new luciferase, NanoLuc (NLuc; Promega Corporation, Madison, WI) bridges this gap in signal intensity. NLuc offers several advantages over Firefly (FLuc) and Renilla (RLuc) luciferases including enhanced stability, significantly smaller size, and >150-fold enhancement in luminescence output (Hall et al., 2012; England et al., 2016).

Furthermore, the substrate for NLuc, furimazine, is more stable and exhibits decreased levels of background activity than the substrate for RLuc, coelenterazine, (Hall et al., 2012; England et al., 2016).

We developed a split luciferase DNA biosensor based on the NanoLuc Binary Technology (NanoBiT; Promega Corporation, Madison, WI) complementation reporter system created for NLuc (Dixon et al., 2016) and catalytically inactive Cas9 (dCas9) from *Streptococcus pyogenes*. Due to the high dissociation constant (K_D_ = 190 μM) and extremely low catalytic activity of the NanoBiT complementation reporter system subunits—termed LgBiT and SmBiT—they must be brought into close proximity in order to reassemble full-length NLuc. Thus, our approach was to direct two dCas9-sgRNA-NanoBiT complexes to two target DNA sites with a specific DNA orientation and spacing. Initially, we achieved a maximum of 8-fold increase in signal in live populations of cells transfected with the biosensor and various target DNA scaffolds compared to populations transfected with the biosensor but no target DNA. Subsequently, we tested the sensitivity of the biosensor on specific endogenous genomic DNA sequences across multiple cell lines and compared the signal-to-background of this approach to a common fluorescence-based method. Finally, we were able to detect single copy genome edits induced by CRISPR-Cas9 with single cell resolution through distinct differences in signal intensity between homozygous mutant and wild-type cells.

## Materials and Methods

### Construction of Directional dCas9-NanoBiT and dCas9-NanoLuc Fusion Proteins

The directional fusion constructs containing the LgBiT and SmBiT of NLuc (Promega Corporation) fused to catalytically inactive Cas9 (D10A and H840A double mutant) were generated using the Gibson Assembly method (New England Biolabs). We used an improved version of the pCDNA3-dCas9 containing two nuclear localization signals, an N-terminal 3× Flag epitope tag and [(GGS)_5_] flexible linker sequences and well as two separate multiple cloning sites at the N- and C-termini of dCas9 (vector map shown in Supplementary Methods S1). The LgBiT and SmBiT were each cloned onto the N- and C-termini of dCas9 using two separate multiple cloning sites in the modified pCDNA3-dCas9 vector (Supplementary Methods S1 for sequences). Overnight N- and C- terminal double restriction digests of sets of flanking restriction sites (XbaI, KpnI) and (NheI, NotI) produced the necessary vector backbones for subsequent Gibson Assembly. LgBiT and SmBiT inserts were ordered as gBlocks Gene Fragments (Integrated DNA Technologies) containing approximately 45 bp homologous sequences with the doubly-digested dCas9 vectors upstream and downstream of the two cut sites. A positive control NLuc-dCas9 fusion construct was created using overlap extension PCR on LgBiT-dCas9 and SmBiT-dCas9 gBlocks to directionally splice the sequences followed by the Gibson Assembly method again using the N-terminal doubly digested dCas9 vector. The four assembled dCas9-NanoBiT constructs, the dCas9-NLuc construct, and pGL4.53 (luc2/PGK) Firefly luciferase vector (Promega Corporation) were separately transformed into 5-alpha Competent *E. coli* (New England Biolabs) using a standard chemical transformation procedure with heat shock at 42°C and transformed *E. coli* were plated on LB plates containing ampicillin at a final concentration of 100 μg/ml. After an 18-h incubation at 37°C, MiniPreps (QIAGEN) were created for a subset of large, well-separated colonies. The selected subset of large colonies was screened for recombinant vector and insert using both diagnostic restriction digests and colony PCR. Clones positive for the four NanoBiT inserts, the full NanoLuc insert, and the luc2 insert using both methods were subsequently sequenced to confirm exact sequences were present.

### Construction of sgRNA Expression Plasmids

The sgRNA expression vector backbone was obtained from Addgene (Addgene #41824) and was linearized using a restriction digest with AflII. Two 19-bp sgRNA target sequences common throughout several genomes but not present in the human genome were selected using CRISPRscan and the UCSC genome browser (Supplementary Methods S2 for sequences). Each sgRNA sequence was incorporated into two 60mer oligonucleotides that contained homologous sequences to the sgRNA expression vector for subsequent Gibson assembly. After oligonucleotide annealing and extension, the PCR-purified (PCR purification kit; QIAGEN) 100 bp dsDNA was inserted into the AflII linearized sgRNA expression vector using Gibson assembly.

### Construction of sgRNA Target Site Vector Scaffolds

Scaffolds containing the two sgRNA target sequences in tandem, inverted, and everted orientations were created using two separate plans. The first plan consisted of a series of overlap extension PCRs on ssDNA oligonucleotides (Integrated DNA Technologies) followed by PCR purification using the MinElute PCR Purification Kit (QIAGEN). The resulting target sequence scaffold oligonucleotides were then subjected to a final amplification with 2× GoTaq Green Master Mix (Promega Corporation) to create poly-dT tails and cloned into the PCR4TOPO vector using the Topo TA Cloning Kit for Sequencing (Invitrogen). The second plan consisted of a series of targeted blunt-end double restriction digests on cloned scaffolds from the first plan, PCR-purification (removing oligonucleotides <∼70 bp) again using the MinElute PCR purification kit (QIAGEN), and re-ligation using excess T4 DNA ligase (New England Biolabs). See Supplementary Methods S3 for sequences.

### Plasmid-Based DNA Biosensor Testing in Live HEK 293T Cells

HEK 293T cells were originally purchased from ATCC and maintained in Dulbecco’s Modified Eagle Medium (Life Technologies) supplemented with 10% FBS and 1x Penicillin/Streptomycin at 37°C under 5% CO_2_. In the first experiment, which sought to determine the optimal molar transfection ratio of LgBiT to SmBiT fusion constructs, 25,000 low passage HEK 293T cells per well were seeded in 96-well white opaque-side microplates (Thermo Fisher Scientific) approximately 20 h before transfection. These cells were then transiently transfected with 100 ng total DNA per well using the Lipofectamine 3000 transient transfection protocol (Invitrogen). Each well was co-transfected with 16.67 ng/well of plasmid expressing each dCas9-NanoBiT fusion construct, 16.67 ng/well of plasmid expressing each of two sgRNAs, 16.67 ng/well of plasmids containing the target sequence, and 16.67 ng/well pMAX-GFP plasmid. With these methods, cells were typically transfected at approximately 90–95% efficiency. We tested various LgBiT:SmBiT molar transfection ratios with the construct in excess being transfected at 16.67 ng/well and the lesser construct being decreased by specific amounts to form desired molar transfection ratios. 33 of the LgBiT + SmBiT wells were transfected with the tandem PAMs 10 bp apart target sequence scaffold and 33 of the LgBiT + SmBiT wells were identically transfected but without any target DNA. For wells that did not reach 100 ng total DNA, pUC19 vector was transfected to make up the difference. In this experiment, signals were measured 24 h post-transfection. In our next experiment, several molar excesses of sgRNA to dCas9-NanoBiT fusion constructs (1:1, 1.2:1, 2:1, 5:1, and 20:1) were delivered to cells using the same method as described above, holding the molar amount of sgRNA constant but decreasing the molar amount of dCas9-NanoBiT fusion proteins. We then held the 20-fold molar excess sgRNA parameter constant and progressively decreased the amount of target DNA transfected, making up the difference with pGL4.53 (luc2/PGK) Firefly luciferase vector (Promega Corporation), essentially random DNA with no binding sites with >5 bp homology with the protospacer of either sgRNA. All fluorescent signals were measured on the SpectraMax M5 Microplate Reader (Molecular Devices) with high PMT sensitivity setting and 100 reads/well before taking any luminescent readings. After adding 25 μL furimazine substrate (Promega Corporation) reconstituted at a 1:19 volumetric ratio with Nano-Glo LCS Dilution Buffer (Promega Corporation) according to the Nano-Glo Live Cell Assay System protocol to each well, luminescent signals were measured on the SpectraMax M5 Microplate Reader with 1 s integration and high PMT sensitivity setting. The ideal delivery parameters were used with the same Lipofectamine 3000 transfection protocol for comparing all orientations of PAM orientation, spacer length, and dCas9-NanoBiT fusion construct pairing.

### Luminescence Microscopy and Image Processing

Transfection experimental setup for microscopy sessions was identical to the setup for microplate reader sessions except that inert pUC19 plasmid was added to the transfection mix to account for the amount of plasmid lost by eliminating target DNA and sgRNAs in background transfection conditions. In addition, an auto-association background condition without target DNA (mouse cell lines transfected with sgRNA to locus 1) was included in the measurements of the non-repetitive region of *MUC4* intron 1 in addition to the no sgRNA background condition as locus 1 sgRNA has no matches with 100% homology within the mouse genome. With these methods, cells were typically transfected at approximately 90–95% efficiency. In these experiments, low-passage HEK 293T, HeLa, MCF7, HCT116, K562, and JLat cells were plated in SensoPlate 24 Well F-Bottom, Glass Bottom Black Microplates (Greiner Bio-One). Next, instead of imaging whole well populations of adherent cells, we split the cells to 1.5 × 10 (Ren et al., 2019) cells/mL and took images of the cell suspensions on Superfrost Plus Microscope Slides (Fisher Scientific) with Premium Cover Glass (Fisher Scientific) combined at a 1:1 volumetric ratio with reconstituted furimazine substrate (Promega Corporation). The Nano-Glo Live Cell Reagent furimazine is nontoxic and nonlytic when delivered to live cells. An optimized NLuc imaging protocol was developed for use on the Leica DM6000 B fully automated upright microscope equipped with the Leica DFC9000 GT sCMOS camera and the Exfo X-Cite 120 Fluorescence Illumination System in which cells were placed in a dark box with all light sources blocked and lamp intensity was set to 0, exposure time was set to 30 s, and sCMOS gain was set to 2.0. The GFP signal produced from pMAX-GFP transfection was imaged using an exposure time of 150 ms and sCMOS gain of 1.0.

Post-processing was applied to better visualize the images. Raw 16-bit grayscale GFP images were recolored using the “green” look up table (LUT), brightness was increased by 50%, and contrast was decreased by 50% in Fiji. Raw 16-bit grayscale NLuc images were recolored using the “red hot” LUT, which displays areas of highest intensity as white and areas of lower or average intensity as varying shades of red. Then, brightness was increased to 100% and contrast was decreased by approximately 50%. Subsequently, the subtract background function was applied in Fiji (Image J) with radius 5.0 and “create background (don’t subtract)” option applied to reduce diffuse background and artifacts from the imaging process. To merge GFP and NLuc images, we directly merged color channels in Fiji (Image J).

Quantitation was then performed on the original unprocessed images. The WEKA Segmentation package (Arganda-Carreras et al., 2017) in Fiji (Image J) was used to segment cells using 50 ROI traces of the nuclear NLuc signals and 50 ROI traces of the background outside of cells as a training data set for nuclear boundaries. This trained WEKA segmentation model was then applied to each NLuc image to determine boundaries of nuclei. Each 8-bit segmented image outputted from the WEKA model was binarized using the auto-threshold function, and the analyze particles function was applied to create ROIs for each nuclear area. These ROIs were then overlaid onto the original unprocessed images. Then, the mean intensity (equivalent to integrated intensity of each nucleus divided by area of each nucleus) after 30 s of total light collection was calculated and recorded for each segmented nuclear area using ImageJ (Fiji). Any cells that were positive for GFP signal but negative for NLuc signal were omitted from final statistical analysis.

### Statistical Testing

Two-tailed student’s t-tests for signal-to-background analyses were conducted in Microsoft Excel 2016. Two-way ANOVA and pairwise Tukey’s HSD post-hoc tests were conducted in R (version 4.0.3) on combinatorial signals from initial biosensing experiments in live cells. Statistics shown in all box-and-whisker plots were computed in R (version 4.0.3).

## Results

### Construction and Optimization of a Split Luciferase DNA Sequence Biosensor

**FIGURE 1:**
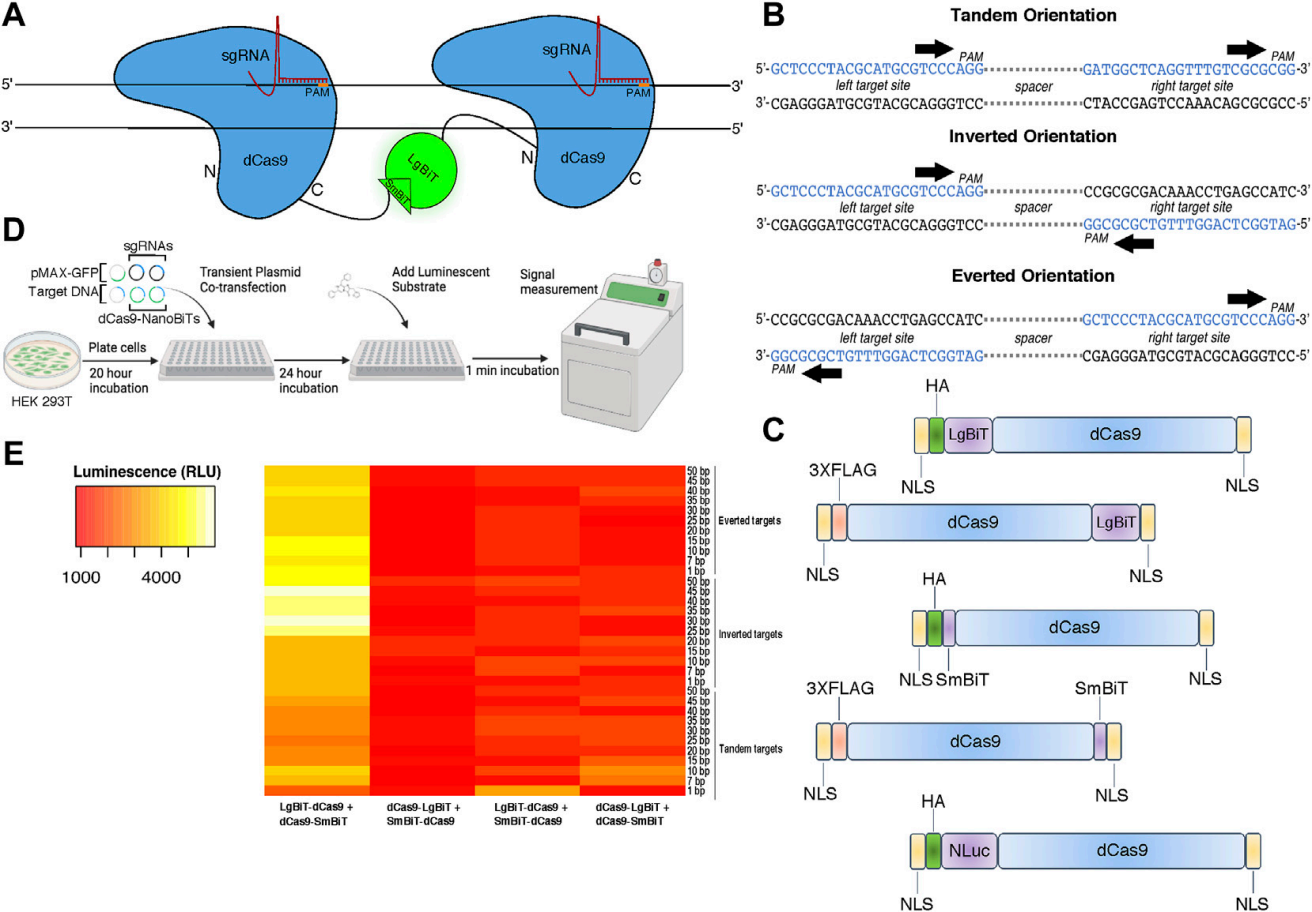
Design and characterization of a split luciferase DNA biosensor. **(A)** A cartoon depiction of sequence-dependent reassembly of NanoLuc luciferase. **(B)** Schematic of target site designs with PAM sites in tandem (parallel on the same strand), inverted (PAMs oriented inward on opposite strands) and everted (PAMs oriented outward on opposite strands). **(C)** Cartoon representation of dCas9-NanoBiT and full-length dCas9-NanoLuc fusion constructs. **(D)** Depiction of experimental process for initial luminometer-based DNA biosensing assays labeling all co-transfected plasmids. **(E)** A heat map showing variation in luminescent signal intensity between four possible orientations of dCas9-NanoBiT fusion proteins across 33 DNA target site spacings and orientations. Sequential scale ranges from lowest signals of the set (red) to highest signals of the set (white).

To design a live cell DNA sequence biosensor, we fused two independently optimized protein fragments of NLuc—LgBiT and SmBiT—to a catalytically inactive Cas9 from *S. pyogenes* (dCas9) (Figure 1A). We constructed five fusion protein plasmids: two in which the LgBiT and SmBiT were fused to the carboxy-terminus of dCas9 (dCas9-LgBiT and dCas9-SmBiT), two in which they were fused to its amino-terminus (LgBiT-dCas9 and SmBiT-dCas9) and one in which full-length NLuc was fused to the amino-terminus of dCas9 (NLuc-dCas9) (Figure 1C; Supplementary Methods S1). For target sites, we produced 33 plasmids each harboring one copy of a DNA target site scaffold containing two SpCas9 sgRNA target sites in three orientations with 1–50 base pair (bp) spacer sequences between them in tandem, inverted, and everted orientations (Figure 1B; Supplementary Methods S2). The two sgRNAs were chosen to have no homology within the human genome and minimal off-targets (Supplementary Methods S3). To determine optimal conditions for this biosensor, different molar ratios of dCas9- and sgRNA-expressing plasmids and different molar amounts of target DNA plasmids were transiently transfected into HEK 293T cells with a range of incubation times post-transfection (Figure 1D). A common reporter for transfection efficiency, pMAX-GFP, was co-transfected in all conditions. Signal-to-background peaked when we used a 10:1 ratio of LgBiT-dCas9 to dCas9-SmBiT fusion proteins, a 20:1 ratio of sgRNA:total NanoBiT plasmid, and a 24-h incubation time between transfection and signal measurement (Supplementary Figure S1). In addition, we found very little dependence of signal-to-background on molar amount of target DNA transfected (Supplementary Figure S1). Hypothesizing that fusion protein orientation and target DNA orientation might have a synergistic effect on signal output, we conducted a two-way ANOVA assuming there was an interaction between these two variables. Significant variation in the efficiency of NLuc reassembly was observed across conditions (Figure 1E), with fusion protein orientation and target DNA orientation being associated with significant differences in luminescent signal output (*p* < 0.0001 and *p* < 0.05, respectively, two-way ANOVA, Supplementary Table S1). The relationship between signal output and fusion protein orientation was also shown to depend on target DNA orientation and vice versa (F (96, 264) = 2.064, *p* < 0.0001, two-way ANOVA, Supplementary Table S1) indicating that these results are affected by an interaction between fusion protein and target DNA orientations. In addition, the LgBiT-dCas9 + dCas9-SmBiT protein configuration produced the highest set of luminescent signals (*p* < 0.0001 for three pairwise comparisons, Tukey HSD, Supplementary Table S2).

### Live Single-Cell Imaging of Repetitive and Unique Endogenous Genomic Sequences

**FIGURE 2:**
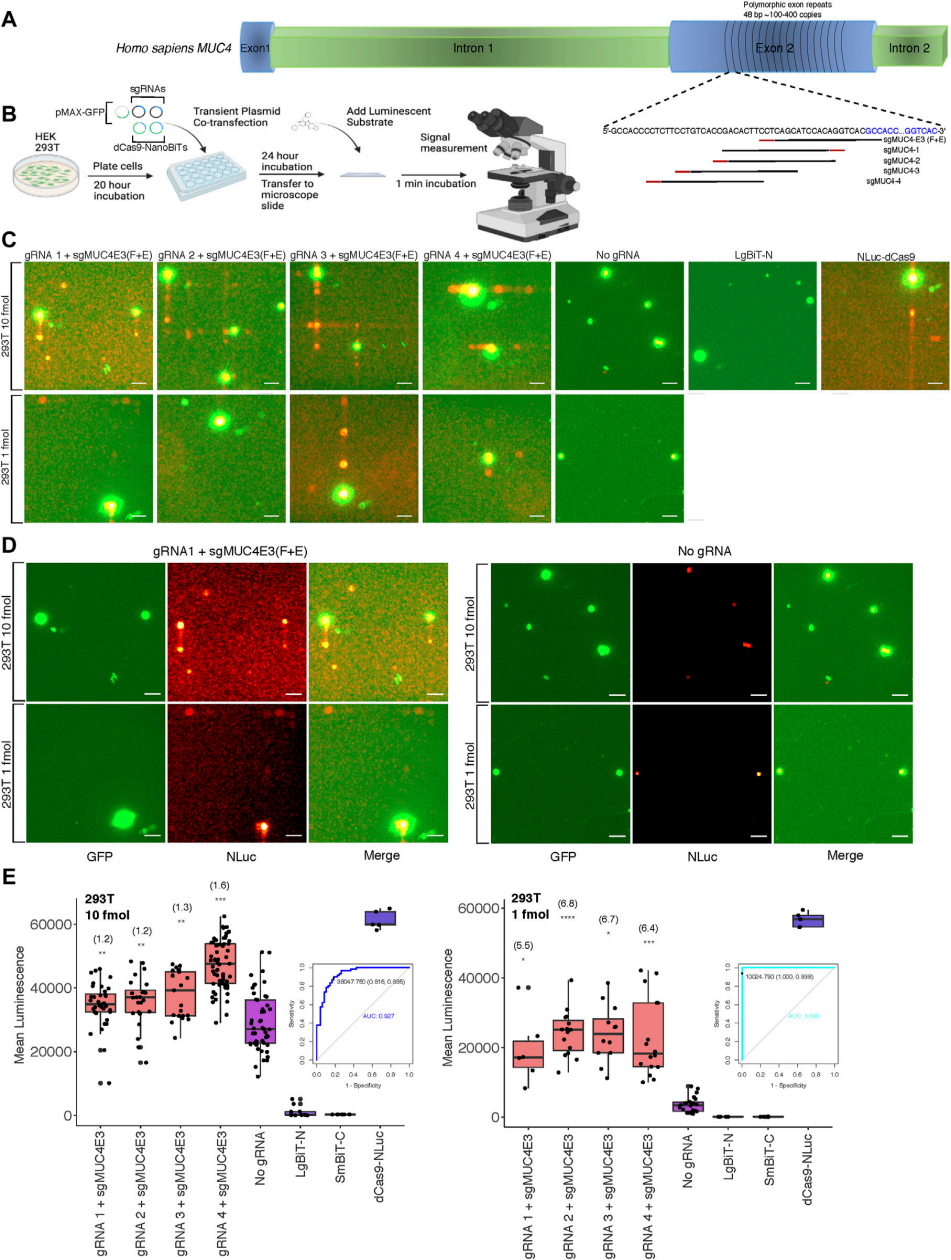
Biosensing repetitive genomic sequences at the human *MUC4* locus **(A)** Cartoon visualization of the repetitive region of exon 2 within the human *MUC4* locus showing sgRNA design strategy using the binding site for sgMUC4-E3(F + E) as an anchor point. **(B)** Depiction of experimental process for all live cell DNA biosensing assays conducted using microscopy, labeling all co-transfected plasmids. **(C)** Merged GFP fluorescence (green) and NLuc luminescence (red) images taken on the Leica DM6000B upright microscope at 10 × magnification depicting dCas9-NanoBiT biosensing of the repetitive region of *MUC4* exon 2 in live HEK 293T cells using two different amounts of dCas9-NanoBiT plasmids in transfection (10 and 1 fmol). Scale bars = 50 μM. **(D)** Individual GFP fluorescence (green), NLuc luminescence (red), and merged images taken on the Leica DM6000B upright microscope at 10 × magnification depicting dCas9-NanoBiT biosensing using sgRNA 1 paired with sgMUC4E3(F + E) at the repetitive region of *MUC4* exon 2 compared to no sgRNA controls using two different amounts of dCas9-NanoBiT plasmids in transfection (10 and 1 fmol). Scale bars = 50 μM. **(E)** Signal quantification for images taken of the split luciferase probe binding the repetitive region of *MUC4* exon 2 in live HEK 293T cells. Apparent signal-to-background ratios are listed in parentheses above each biosensing condition. 5 < *n* < 61, where *n* represents the number of unique cells quantified; unpaired two-sided Student′s t-test, *p < 0.05; **p < 0.01; ***p < 0.001; ****p < 0.0001. Boxes show the median and interquartile range (IQR) and whiskers show dispersion from the IQR that is equal to the lesser of the 1^st^ or 3^rd^ quartiles plus 1.5xIQR or the distance from the 1^st^ or 3^rd^ quartiles to the minimum or maximum points, respectively. Receiver Operating Characteristic (ROC) curves representing biosensing results using sgRNA 4 paired with sgMUC4E3(F + E) at 10 and 1 fmol transfected within the repetitive region of *MUC4* in HEK 293T are shown. False positives were determined by signals due to auto-assembly (no sgRNA). The signal threshold for distinguishing true positives from false positives that maximized Youden’s J Statistic (sensitivity + specificity – 1) is shown as a point on the ROC curve along with corresponding specificity and sensitivity values in parentheses.

After optimizing delivery conditions for our DNA sequence biosensor in live cells using a luminometer to measure luminescence across whole well cell populations, we sought to investigate the feasibility of detecting luminescence in single cells on relatively common imaging equipment. To this end, we modified an upright fluorescence microscope to capture the relatively low light intensities associated with NLuc and other luminescent reporters. Cells were placed in a dark box with all light sources blocked and exposure times were lengthened. To determine the applicability of our split luciferase biosensor to imaging endogenous DNA sequences, we first compared its sensitivity to that of both a previously described dCas9-EGFP fluorescent probe (Chen et al., 2013) and the NLuc-dCas9 probe from our study. We used a single optimized sgRNA, sgMUC4-E3 (F + E) (Chen et al., 2013) to direct these probes to bind a region of polymorphic 48-bp repeats of copy number between approximately 100 and 400 within exon 2 of the human *MUC4* locus (Figure 2A). We found that both full reporter probes had comparable signals when binding the tandem repeats compared to a background condition with no sgRNA in HEK 293T and HeLa cells (Supplementary Figure S2). We then used sgMUC4-E3(F + E) as an anchor sgRNA and constructed four sgRNAs with unique spacer lengths and orientations around it to direct our split luciferase probe to bind the same repetitive region of *MUC4* in HEK 293T cells (Supplementary Methods S4 for target sequences and construction methods). Subsequently, we compared signals of pairwise combinations of each the four unique sgRNAs and sgMUC4(F + E) to signals of an identical transfection without gRNA in HEK 293T cells. We observed variable sensitivity for the *MUC4* tandem repeats based on molar amount of probe transfected and target site configuration (Figures 2B–E). Overall gRNA-directed signal was greater at higher concentrations of probe, but with accompanying greater background signal in cells lacking gRNA. However, we found that signal-to-background was greatly improved to approximately 5.5-7-fold by reducing the amount of probe delivered from 10 to 1 fmol. To assess the diagnostic power of our probe to detect true positives for presence of a single repetitive genomic locus, we used Receiver Operating Characteristic (ROC) analysis. We found that sensitivity and specificity of the probe increased at lower concentrations in transfection, from ∼0.89 to ∼0.94 and from ∼0.82 to 1.0, respectively. (Figures 2B–E).

**FIGURE 3:**
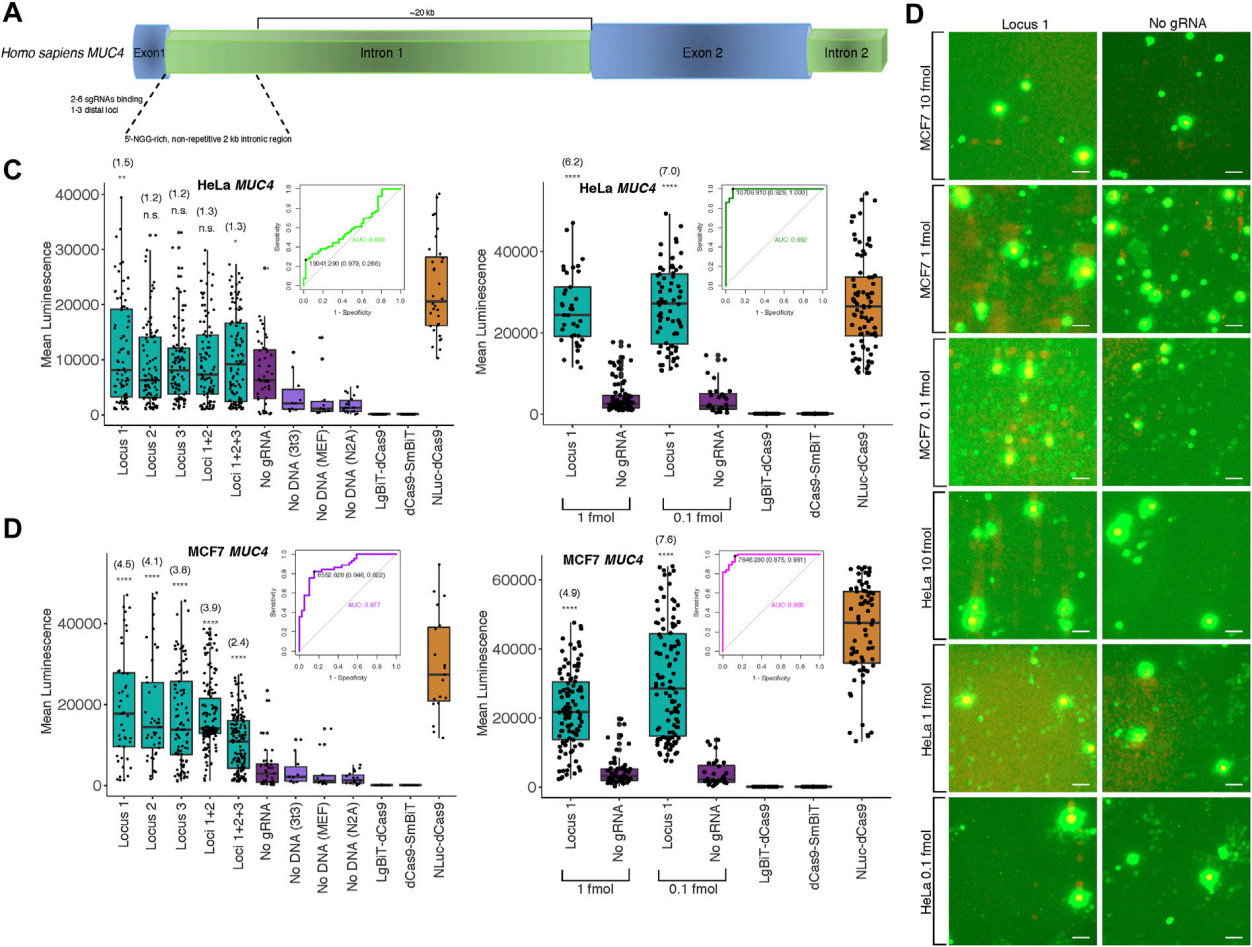
Biosensing non-repetitive genomic sequences at the human *MUC4* locus **(A)** Cartoon visualization of the non-repetitive region within intron 1 of human *MUC4* showing sgRNA design strategy. **(B)** Merged fluorescence (green) and luminescence (red) images taken on the Leica DM6000B upright microscope at 10 × magnification depicting dCas9-NanoBiT biosensing of a single locus within non-repetitive *MUC4* intron 1 in live MCF7 and HeLa cells. Scale bars = 50 μM. **(C)** Signal quantification for images taken of the dCas9-NanoBiT probe binding to several combinations of loci in the non-repetitive region of *MUC4* intron 1 in HeLa cells at 10 fmol probe transfected (left) and for images taken of the probe binding to a single non-repetitive locus within *MUC4* intron 1 at 1 and 0.1 fmol probe transfected in HeLa cells (right). Apparent signal-to-background ratios (no sgRNA = background condition) are listed in parentheses. 32 < *n* < 150 for HeLa cells at 10 fmol and 28 < *n* < 100 for HeLa cells at the two lower concentrations, where *n* represents the number of unique cells quantified; unpaired two-sided Student′s *t*-test, *p < 0.05; **p < 0.01; ***p < 0.001; ****p < 0.0001. Receiver Operating Characteristic (ROC) curves representing biosensing results at *MUC4* locus 1 at 10 fmol (left) and 1 fmol (right) probe transfected in HeLa cells are shown. **(D)** Signal quantification for images taken of the dCas9-NanoBiT probe binding to several combinations of loci in the non-repetitive region of *MUC4* intron 1 in MCF7 cells (left) and for images taken of the probe binding to a single non-repetitive locus within *MUC4* intron 1 at two lower concentrations in MCF7 cells (right). Apparent signal-to-background ratios are listed in parentheses. 19 < *n* < 159 for MCF7 cells at 10 fmol and 32 < *n* < 106 for MCF7 cells at the two lower concentrations, where *n* represents the number of unique cells quantified; unpaired two-sided Student′s *t*-test, *p < 0.05; **p < 0.01; ***p < 0.001; ****p < 0.0001. Receiver Operating Characteristic (ROC) curves representing biosensing results at *MUC4* locus 1 at 10 fmol (left) and 1 fmol (right) probe transfected in MCF7 cells are shown. Boxes show median and IQR and whiskers show dispersion from IQR.

Since the majority of loci within the human genome are non-repetitive, a more significant application would be the potential detection of such low copy number, unique genomic sequences. To this end, we targeted the non-repetitive region of intron 1 of the human *MUC4* locus with 1-3 pairs of unique sgRNAs tiling along the locus with at least 200 bp between pairs to avoid interactions between probe components at different binding sites (Figure 3A; Supplementary Figure S3, Supplementary Methods S4 for target sequences and construction methods). We observed strong cell type-specific differences in biosensor sensitivity based on the amount of probe transfected (Figure 3B; Supplementary Figures S3–S5, S8). Specifically, signal-to-background in HeLa cells peaked at approximately 1.3-fold using a single pair of sgRNAs and 10 fmol probe in transfection but at 7-fold using 0.1 fmol probe in transfection. Furthermore, to assess the diagnostic power of our probe to detect true positives for presence of a single non-repetitive genomic locus, we used Receiver Operating Characteristic (ROC) analysis. We found area under the curve (AUC) was 0.608 at 10 fmol probe transfected to HeLa cells, whereas AUC increased to 0.992 when 1 fmol was transfected (Figure 3C). Likewise, using a single pair of sgRNAs and 10 fmol probe in MCF7 cells, signal-to-background peaked at 4.5-fold, but was increased to 7.6-fold by reducing amount of probe transfected to 0.1 fmol. Whereas AUC was 0.877 at 10 fmol transfected to MCF7 cells, AUC increased to 0.983 when 1 fmol was transfected (Figure 3D). At 10 fmol probe transfected in HCT116, K562, 293T, and Jlat cells, AUC was 0.622, 0.734, 0.841, and 0.856, respectively (Supplementary Figure S3C).

#### Live Single-Cell Biosensor Imaging of Single-Base Changes Induced by CRISPR-Cas9 Editing

**FIGURE 4:**
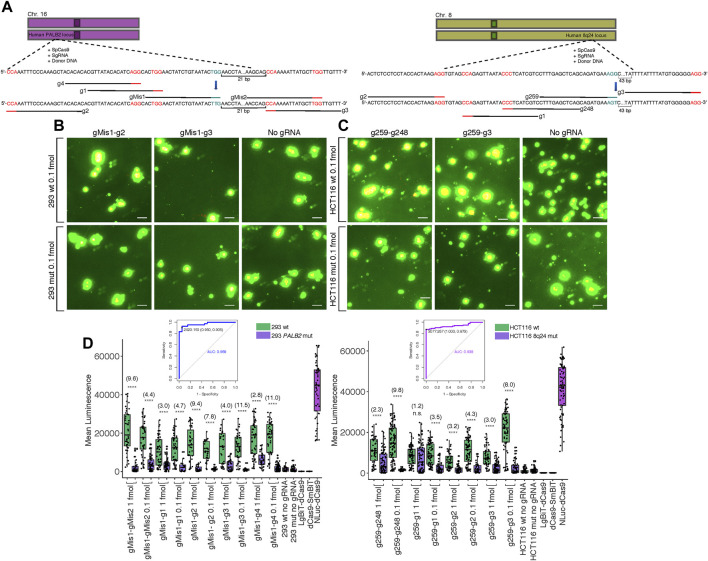
Biosensing CRISPR-Cas-induced genome edits in live cells. **(A)** Cartoon visualization of the CRISPR-Cas editing experiments conducted at the human 8q24 poly-cancer risk locus and at *PALB2*. sgRNAs used for editing have blue PAM sites while sgRNAs around the site of mutation used for detection of mutant cells in biosensing experiments have red PAM sites. Single base pair edits are shown in bold. **(B)** Merged fluorescence (green) and luminescence (red) images taken on the Leica DM6000B upright microscope at 10 × magnification of the dCas9-NanoBiT probe applied to the *PALB2* locus in wild-type and confirmed homozygous mutant cells after targeted CRISPRCas9 genome editing. Scale bars = 50 μM. **(C)** Merged images taken on the Leica DM6000B upright microscope at 10 × magnification of the dCas9-NanoBiT probe applied to the 8q24 poly-cancer risk locus in wild-type and confirmed homozygous mutant cells after targeted CRISPR-Cas9 genome editing. Scale bars = 50 μM. **(D)** Signal quantification for the above images as well as several other sgRNA pairs. Apparent signal-to-background ratios (mutant cell signal = background) are listed in parentheses. Data are presented such that boxes show the median and interquartile range (IQR) and whiskers show dispersion from the IQR that is equal to either the 1^st^ or 3^rd^ quartiles minus/plus 1.5xIQR or the distance from the 1^st^ or 3^rd^ quartiles to the minimum or maximum points, respectively. 26 < *n* < 55 for 293 wild-type, 20 < *n* < 84 for 293 mutant, 51 < *n* < 102 for HCT116 wild-type, and 32 < *n* < 86 for HCT116 mutant, where *n* represents the number of unique cells quantified; unpaired two-sided Student′s t-test, *p < 0.05; **p < 0.01; ***p < 0.001; ****p < 0.0001. Receiver Operating Characteristic (ROC) curves representing biosensing results within CRISPR-Cas edited lines are shown. For 293 cells, the ROC represents sgRNA pair gMis1-g3 and 0.1 fmol probe delivered and for HCT116 cells, the ROC represents sgRNA pair g259-g248 and 0.1 fmol probe delivered. False positives were determined using signals in homozygous mutant lines.

One of the most pertinent applications for our split luciferase biosensor is the detection of various mutations in genomic DNA sequence after targeted genome editing with CRISPR-Cas9. Thus, we created G > T missense single nucleotide polymorphisms (SNPs) at two different loci in two cell lines: within the 8q24 multi-cancer risk locus in HCT116 cells and within the PALB2 locus in HEK 293 cells (Figure 4A). Both SNPs were present within the PAM site of the sgRNA used for editing (Coggins et al., 2017) (Supplementary Methods S5). In a previous study, mutants were confirmed to be homozygous for the G > T mutations by dilution plating followed by detection of specific alleles by Kompetitive Allele-Specific PCR (KASP) in expanded populations (Coggins et al., 2017). We hypothesized that these mutations should completely inhibit binding by the sgRNA used for editing, making luminescence within mutant cell clones lower than luminescence within wild-type cell clones transfected with the same probe components. To investigate this hypothesis, we transfected wild-type and homozygous mutant clones of both cell lines with the dCas9-NanoBiT probe carrying the sgRNA used for editing along with 4-5 sgRNAs flanking it in various orientations at various distances. We observed reduced luminescence in the mutant clones compared to the wild-type clones when 0.1 fmol probe was delivered to cells (Figures 4B,C; Supplementary Figures S6, S7).

Specifically, luminescence in the HEK 293 wild-type clones was approximately 2.8–11.5-fold higher across all five sgRNA pairs tested at *PALB2* compared to HEK 293 mutant clones (Figure 4D). Likewise, in HCT116, luminescence in the wild-type clones was approximately 1.2–9.8-fold higher across all four sgRNA pairs tested at the 8q24 poly-cancer risk locus compared to mutant clones (Figure 4D).

## Discussion

Traditionally, fluorescence signal-to-background analysis involves specialized confocal fluorescent microscopes that can resolve bright foci and compare these signals to the background nucleoplasm at high resolution. We envisioned a platform for measurement of luminescence in single cells on relatively common imaging equipment normally geared toward fluorescence. Our hope was that this might reduce the need for expensive, specialized imaging equipment for luminescence and ultimately serve to lower the barriers to entry for this technique. Unlike imaging for traditional fluorescent DNA probes, the imaging resolution of this technique is insufficient to consistently distinguish bright foci from the larger regions of signal accumulation within the nucleoplasm. However, a range of signal intensity across the nuclear regions is visible in many nuclei. It is possible the whiter signal areas represent the approximate location of the probe within the nucleus and the remainder of the signal accumulation area represents free-floating, complemented NanoLuc. In addition, our no sgRNA background condition represents signal resulting from complemented NLuc that is not bound to DNA for a given transfection condition. Thus, we expect differences in our SBR metric are primarily due to total complemented NanoLuc minus free-floating complemented NanoLuc and the majority of signal in on-target conditions should be produced through bright luminescent foci on the DNA.

When optimizing delivery conditions, we found using 20-fold molar excess sgRNA plasmid in transfection compared to dCas9-NanoBiT plasmid resulted in an increase in signal-to-background compared to other tested ratios. This result can partially be explained by the shorter nuclear lifetime of cellular RNAs compared to both cellular DNA and proteins (Schwanhäusser et al., 2011). Since RNA molecules are degraded much faster than their DNA and protein counterparts, transient plasmid transfection-based delivery of this biosensor may require higher initial amounts of DNA template for the sgRNA to reach a steady-state level of transcription in cells. These factors may also explain our ideal incubation time before measurement of luminescence post-transfection of 24 h. Plasmid transcription, mRNA degradation, and mRNA translation show exquisite temporal control in cells (Schwanhäusser et al., 2011), and a 24-h incubation time likely resulted in fairly stable levels of both the dCas9-NanoBiT fusion proteins and available sgRNAs, allowing for high rates of sgRNA-fusion protein association and DNA binding in cells.

In all cases where <10 fmol of probe plasmid was delivered to cells, we observed differences between on-target conditions and background conditions at both repetitive and non-repetitive regions of *MUC4* that were statistically significant (*p* < 0.01 and *p* < 0.0001, respectively, unpaired student’s t-test, two-tailed). These marked differences in signal intensities indicated NLuc reassembly was occurring in target cell nuclei upon binding of the probe to these regions of the *MUC4* locus. In addition, ROC analysis showed our probe to be an excellent discriminator of true and false positive detection events when transfected at low concentrations at endogenous *MUC4* with high area-under-the-curve for both repetitive and non-repetitive sequences. Thus, our split luciferase probe can detect low copy number sequences with high sensitivity and specificity and optimal signal cutoff points can be selected using statistics such as Youden’s J to maximize these parameters. However, given variable signal-to-background ratios across six cell lines tested, the performance of this probe is moderately cell-type specific. A number of factors may be expected to vary to some degree across different cell lines, including transfection efficiency, sgRNA and fusion protein decay rates, uptake efficiency of the luminescent substrate, or attenuation rate of the resulting signal. In addition, we observed a relatively high degree of variability within each transfection condition. Additional optimization of probe design and methodology in future studies, such as fine-tuning linker composition and length and exploring alternatives to transient transfection, could reduce variability, increase complementation efficiency, and improve the utility of this approach. The copy number of a given genomic locus may also vary across cell lines, with higher copy numbers potentially resulting in more robust signal output.

In addition, we envisioned that we could apply our probe to isolate mutant cells from a population of genome-edited cells via detection of SNPs induced by editing experiments at two genomic loci in HEK 293 and HCT116 cells. We found that luminescent signals were higher across several sites bound by sgRNA pairs around the original Cas9 cut site in wild-type HEK 293 and HCT116 cells compared to homozygous mutant HEK 293 and HCT116 cells. This effectively demonstrated differentiation between binding two and zero copies of the target sequence, as HEK 293 cells have two copies of chromosome 16 and HCT116 cells have two copies of chromosome 8 with no commonly reported abnormalities (Roschke et al., 2002; Lin et al., 2014). We hypothesized that mutating the PAM site in both cell lines would create a condition where Cas9 would not be able to recognize the original target site (Jiang and Doudna, 2017). The fact that all sgRNA pairs produced higher signals in wild-type compared to mutant cells shows that our split luciferase probe can detect very minor differences in genomes across individual cells, including differences in SNP copy number. For mutant cells to be expected to glow brighter than wild-type so desired mutants can be more easily isolated in practice, guide RNA design for the probe could be specifically altered. In addition, ROC analysis of this data showed our probe to be an excellent discriminator of true and false positives for SNP detection with high area-under-the-curve for edits at both *PALB2* and 8q24. Thus, our probe can also detect SNPs with high sensitivity and specificity. However, it is worth noting that mismatches within the PAM site are less permissive to binding than those within the sgRNA hybridization region. SNPs occurring in the protospacer region should be evaluated for their effect on specificity of the probe. For screening edited cells from wild-type cells within an edited cell population, perhaps the most pertinent metric is the minimum fraction of edited cells required to be screened in order to make a positive call. This is also equal to 1 minus the probability of correctly identifying a positive clone within the mutant cell population or 1—sensitivity. For the 293 edited cells, since we observed a sensitivity of ∼0.91 within an isogenic mutant population, we would need to screen a minimum of 9% of cells within this population to detect a true positive mutant clone. Likewise, for the HCT116 edited cells, since we observed a sensitivity of ∼0.88, we would need to screen a minimum of 12% of cells within this population to find a true positive mutant clone. In practice, the minimum fraction of cells required to be screened from an edited cell population would be much higher, as this population is a mixture of both homozygotes and heterozygotes and the ratio of mutant to wild-type clones would depend on the editing efficiency.

In practice, signal detection with this probe could potentially be used as a precursor to manual single cell isolation after gene editing, which could allow users of gene editing techniques to save valuable time and resources during the single cell cloning process. As we transfect at a high density and replate cells at a low density for imaging, this technique sets the stage for the next step, which would be to isolate the cells with the detected edit. It is conceivable that after transfecting an edited cell population with our probe and taking a subset of this edited population for imaging, single mutant cells could be isolated by manual separation using specialized cloning cylinders based on differences in luminescent signal intensity. Then, these isolates could be clonally expanded to produce an isogenic cell population. We conclude that this split luciferase probe should be a broadly useful platform for many live cell DNA biosensing applications that require low copy number resolution and minimal destruction of highly valuable cell populations, including identification and isolation of mutant cells from a population of cells that has undergone a genome editing procedure, real-time identification of cells harboring new driver mutations or broad chromosomal rearrangements such as inversions or translocations during neoplasia, or more generally, *in situ* genotyping of heterozygotes and homozygotes at a defined locus.

## Data Availability

The original contributions presented in the study are included in the article/Supplementary Material, further inquiries can be directed to the corresponding author.
