# Supplementary material for: Imaging Unique DNA Sequences in Individual Cells Using a CRISPR-Cas9-Based, Split Luciferase Biosensor
Source: Front Genome Ed. 2022 Mar 25;4:867390. doi: 10.3389/fgeed.2022.867390 (PMC8990833; doi:10.3389/fgeed.2022.867390)
Supplement: Supplementary file 1 [file DataSheet1.PDF]

SUPPLEMENTARY INFORMATION
for
Imaging Unique DNA Sequences in Individual Cells Using a CRISPR-Cas9-Based, Split Luciferase Biosensor

Nicholas G. Heath[1,2,3], Henriette O'Geen[1,3], Nicole B. Halmai[1,3], Jacob Corn[3,4] & David J. Segal[1,2,3*]

[1]Genome Center and Department of Biochemistry and Molecular Medicine, University of California, Davis, Davis, CA, 95616, United States

[2]Integrative Genetics and Genomics, University of California, Davis, Davis, CA, 95616, United States

[3]Innovative Genomics Institute, University of California, Berkeley, CA, 94720, United States

[4]Institute for Molecular Health Sciences, ETH, Zurich, Switzerland

*To whom correspondence should be addressed: djsegal@ucdavis.edu

Figure S1: Optimization of plasmid-based delivery………………………………………. 2-3

Figure S2: Signal-to-background of full reporter probes….………………………………4

Figure S3: dCas9-NanoBIT biosensing of nonrepetitive sequences at *MUC4* in six live cell lines………………………………………………………………………………...5-6

Figure S4: dCas9-NanoBIT biosensing of a single nonrepetitive locus at *MUC4* at reduced probe concentrations in MCF7 cells…………………………………………………………7

Figure S5: dCas9-NanoBIT biosensing of a single nonrepetitive locus at *MUC4* at reduced probe concentrations in HeLa cells…………………………………………………………8

Figure S6: dCas9-NanoBIT biosensing of CRISPR-induced SNP at PALB2 locus in HEK 293 cells…………………………………………………………………………... 9

Figure S7: dCas9-NanoBIT biosensing of CRISPR-induced SNP at 8q24 poly-cancer risk locus in HCT116 cells……………………………………………………………………..10

Figure S8: dCas9-NanoBIT biosensing at nonrepetitive MUC4…………………………...11

Table S1 and Table S2……………………………………………………………………..12

Extended experimental procedures………………………………………………………13-35

Fig. S1: Optimization of plasmid-based delivery

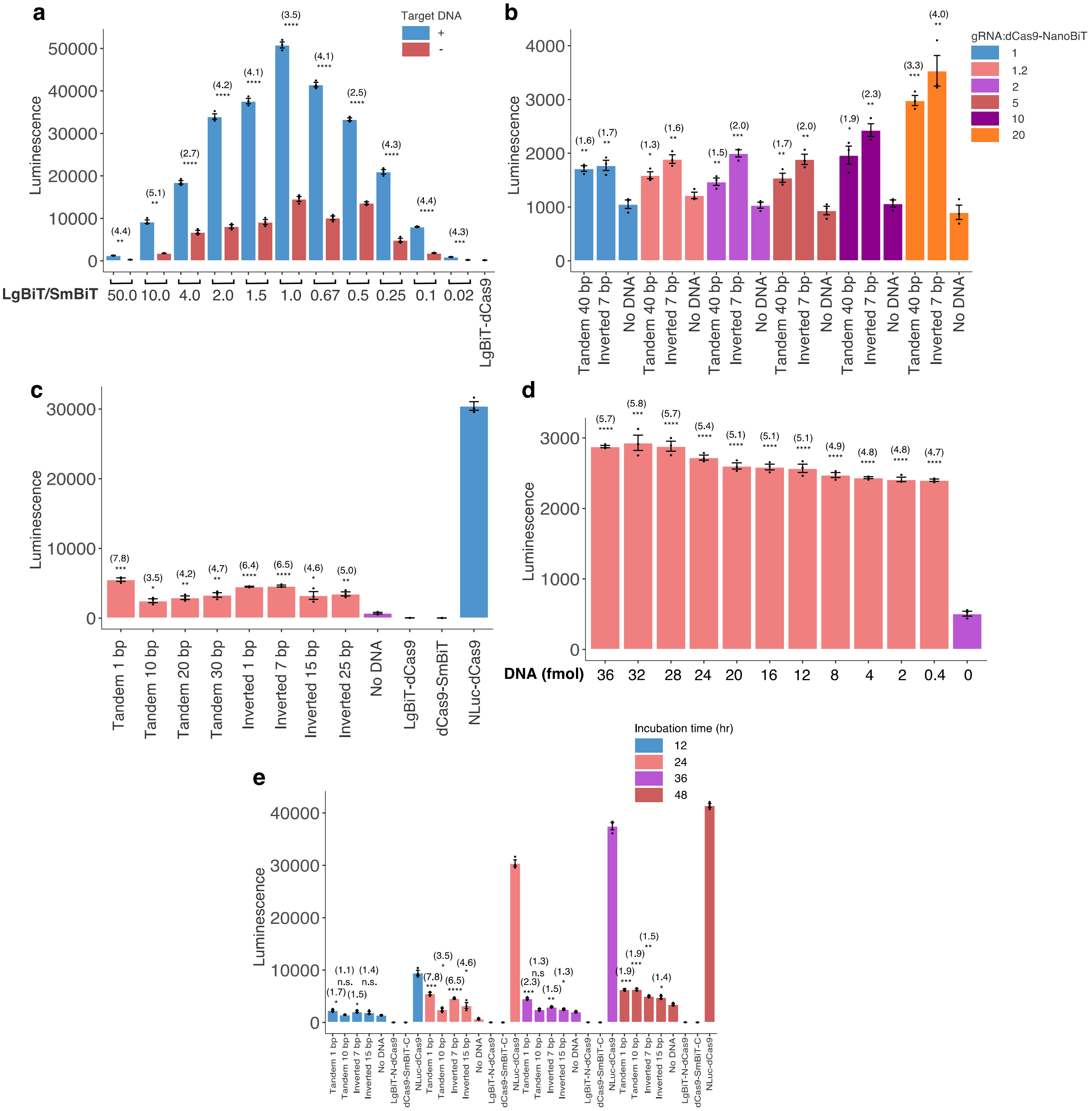

Fig. S1: (a) To determine the optimal ratio of LgBiT:SmBiT in terms of signal production, plasmids expressing LgBiT-dCas9 and dCas9-SmBiT fusion proteins were co-transfected in ratios ranging between 50:1 and 1:50 with or without target DNA plasmids. For simplicity, this initial experiment used only tandemly orientated target sites with 10-bp spacers, as this design was expected to bring the luciferase subunits into close proximity based on the idea that spacers that are a multiple of the helical turn length should foster target site alignment on the double helix. Relative NLuc signal intensity is shown across indicated molar transfection ratios of LgBiT-dCas9 to dCas9-SmBiT with (blue bars) or without (red bars) DNA target plasmids in HEK 293T cells. **(b)** Signal intensities of tandem 40-bp and inverted 7-bp DNA targets compared to no DNA controls over 1:1, 1:1.2, 1:2, 1:5, 1:10, and 1:20 fusion protein:gRNA molar transfection ratios. **(c)** Relative signal intensities using targets of indicated spacing and orientation. gRNAs plasmids were transfected at 20-fold molar excess to dCas9-NanoBiT fusion constructs. **(d)** The dependence of target plasmid concentration was assayed using fixed ratios of the dCas9-NanoBiT and gRNA plasmids. **(e)** The dependence of incubation time post-transfection was assayed using fixed ratios of all plasmids in the indicated configurations. Apparent signal-to-noise ratios in **a-e** (comparisons made to no DNA background conditions) are listed in parentheses above each biosensing condition. Data in **a-e** are presented as the mean ± s.e.m., $n = 3$, where n represents the number of independent experimental technical replicates included in parallel; unpaired two-sided Student′s t-test, *$P < 0.05$; **$P < 0.01$; ***$P < 0.001$; ****$P < 0.0001$. In transfections where the amount of one dCas9-NanoBiT interaction partner was decreased, an equal amount of inert pUC19 DNA was included in the transfection mix.

Fig. S2: Signal-to-background of full reporter probes

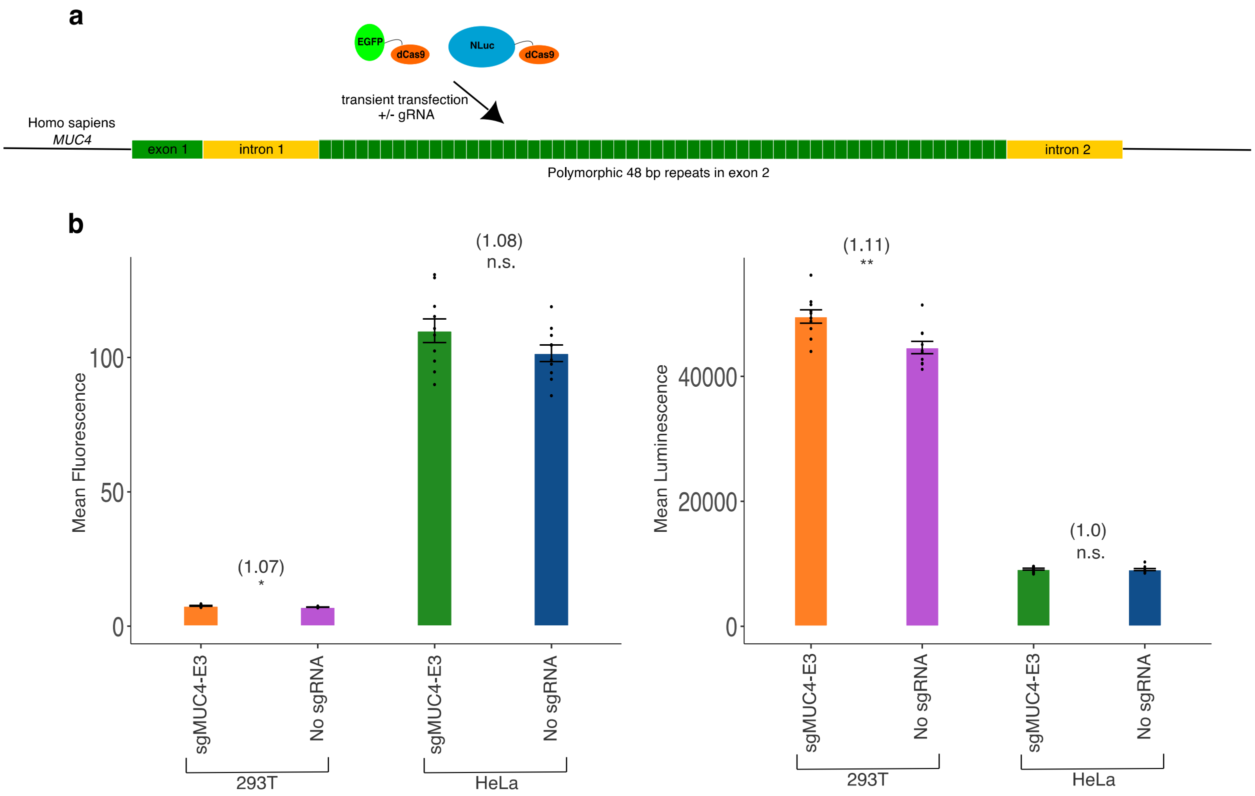

Fig. S2: (a) Cartoon representation of a transient transfection experiment to directly compare signal-to-noise of full reporter probes to split reporter probes (background condition without sgRNA transfected as reported in the main text). Experimental conditions are identical to those in Fig. 2 targeting the non-repetitive region of exon 2 within the human *MUC4* locus. **(b)** On-target signal compared to no gRNA background conditions for dCas9-EGFP fluorescent probe shown in two cell lines. **(c)** On-target signal compared to no gRNA background conditions for NLuc-dCas9 luminescent probe shown in two cell lines. Apparent signal-to-noise ratios in **a-b** (comparisons made to no sgRNA background conditions) are listed in parentheses. Data in **a-b** are presented as the mean ± s.e.m., $n = 10$, where n represents where n represents the number of unique cells quantified; unpaired two-sided Student's t-test, $*P < 0.05$; $**P < 0.01$; $***P < 0.001$; $****P < 0.0001$, n.s. = not significant.

Figure S3: dCas9-NanoBiT Biosensing of Nonrepetitive Sequences at *MUC4* In Six Live Cell Lines

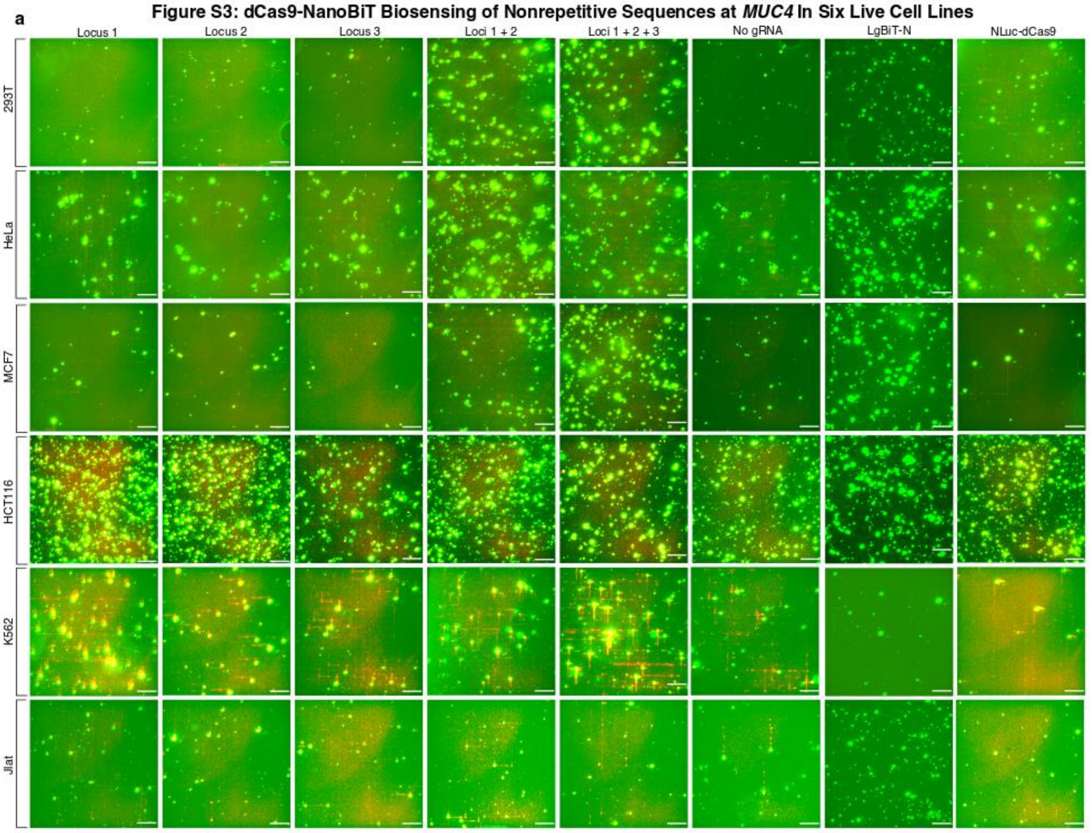

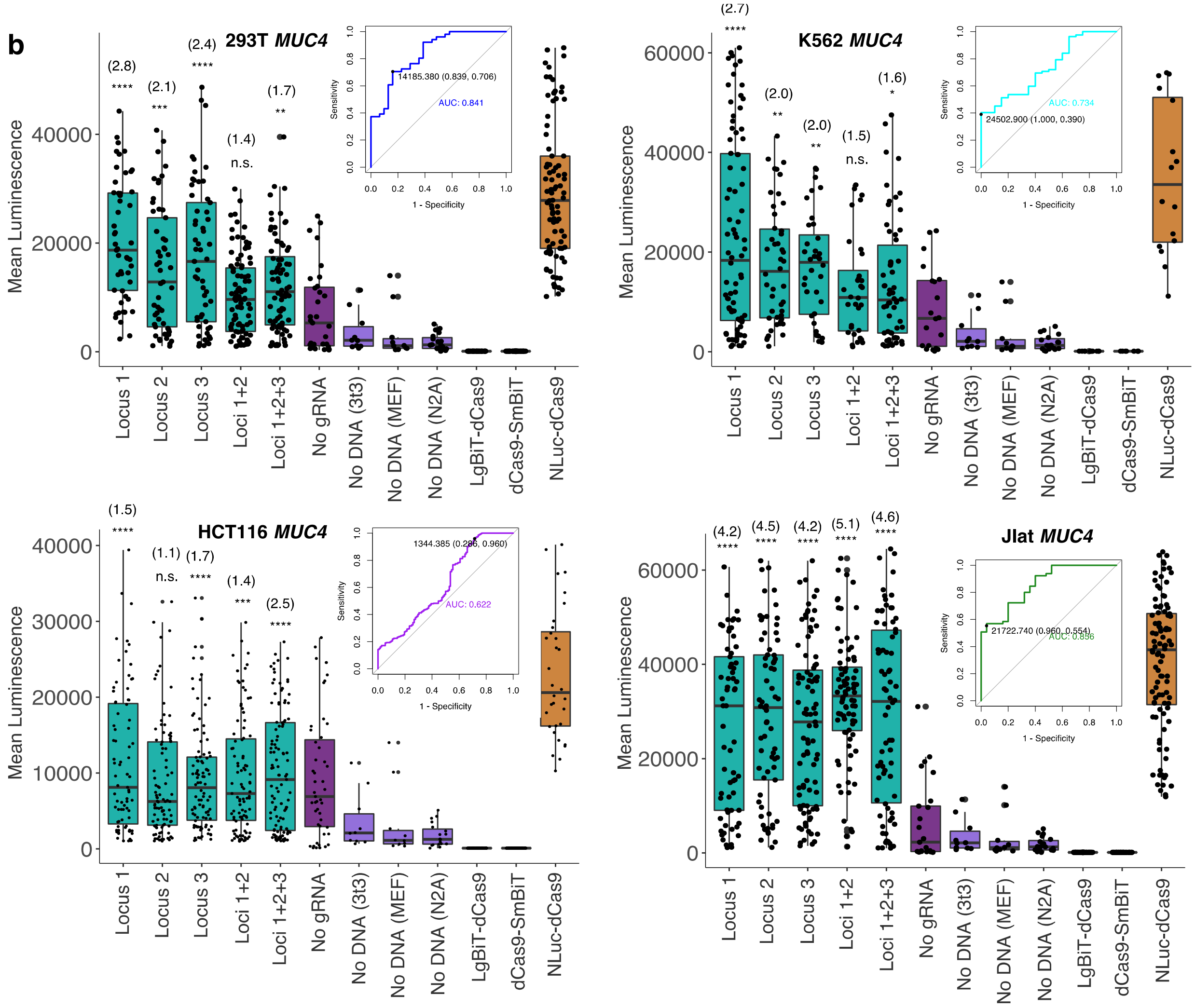

Fig. S3: dCas9-NanoBiT biosensing images of three loci individually and in combinations of two and three within the nonrepetitive region of intron 1 of the *MUC4* gene in six human cell lines at 10 fmol probe transfected. The two suspension lines (Jlat and K562) were electroporated with the Invitrogen Neon Transfection System and the other four adherent lines were transfected with Lipofectamine 3000. **(a)** Panel of images representing merged GFP and NLuc channels at 10X magnification taken on the Leica DM6000 B upright microscope. Scale bars=200 µM. **(b)** Signal quantification of above images for four cell lines not displayed in the main text. Apparent signal-to-noise ratios (comparisons made to no sgRNA background conditions) are listed in parentheses above each biosensing condition. Error bars represent s.e.m., with $31 < n < 94$ for 293T cells, $112 < n < 439$ for HCT116 cells, $8 < n < 82$ for K562 cells, and $25 < n < 106$ for Jlat cells, where n represents the number of unique cells quantified; unpaired two-sided Student's *t*-test, *$p < 0.05$; **$p < 0.01$; ***$p < 0.001$; ****$p < 0.0001$. Receiver Operating Characteristic (ROC) curves representing biosensing results at locus 1 within *MUC4* in all four additional cell lines are shown. False positives were determined by signals due to auto-assembly (no sgRNA). The signal threshold for distinguishing true positives from false positives that maximized Youden's J Statistic (sensitivity + specificity – 1) is shown as a point on the ROC curve along with corresponding specificity and sensitivity values for this threshold in parentheses.

Fig. S4: dCas9-NanoBiT Biosensing of a Single Nonrepetitive Locus at *MUC4* at Reduced Probe Concentrations in MCF7 Cells

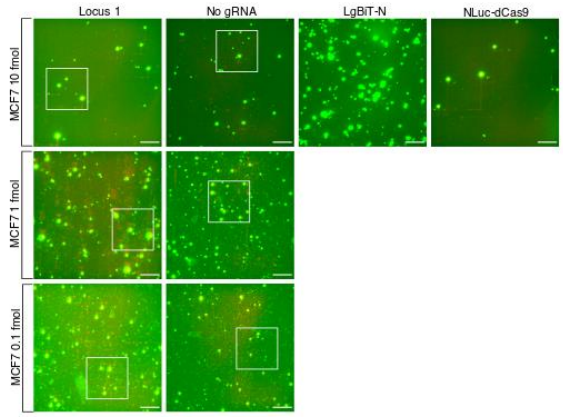

Fig. S4: Panel of images depicting merged GFP (fluorescence) and NLuc (luminescence) channels at 10X magnification taken on the Leica DM6000 B upright microscope. Images represent dCas9-NanoBiT biosensing of a single locus within the nonrepetitive region of intron 1 of the *MUC4* gene in MCF7 cells at all three amounts tested for transfection (10 fmol, 1 fmol, and 0.1 fmol). Controls with no gRNA transfected, LgBiT-dCas9 alone transfected, and NLuc-dCas9 probe transfected are shown for comparison. Scale bars=200 µM. Boxes are shown in the above images which depict approximately 437 µM x 437 µM square sections and represent the image sections shown in Fig. 3b.

Fig. S5: dCas9-NanoBiT Biosensing of a Single Nonrepetitive Locus at *MUC4* at Reduced Probe Concentrations in HeLa Cells

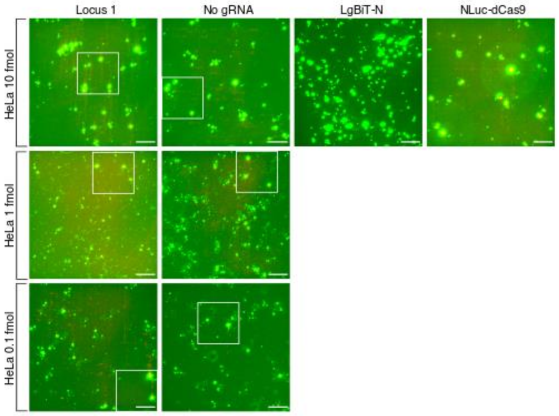

Fig. S5: Panel of images depicting merged GFP (fluorescence) and NLuc (luminescence) channels at 10X magnification taken on the Leica DM6000 B upright microscope. Images represent dCas9-NanoBiT biosensing of a single locus within the nonrepetitive region of intron 1 of the *MUC4* gene in HeLa cells at all three amounts tested for transfection (10 fmol, 1 fmol, and 0.1 fmol). Controls with no gRNA transfected, LgBiT-dCas9 alone transfected, and NLuc-dCas9 probe transfected are shown for comparison. Scale bars=200 µM. Boxes are shown in the above images which depict approximately 437 µM x 437 µM square sections and represent the image sections shown in Fig. 3b.

Fig. S6: dCas9-NanoBiT Biosensing of CRISPR-Induced SNP at *PALB2* Locus in HEK 293 Cells

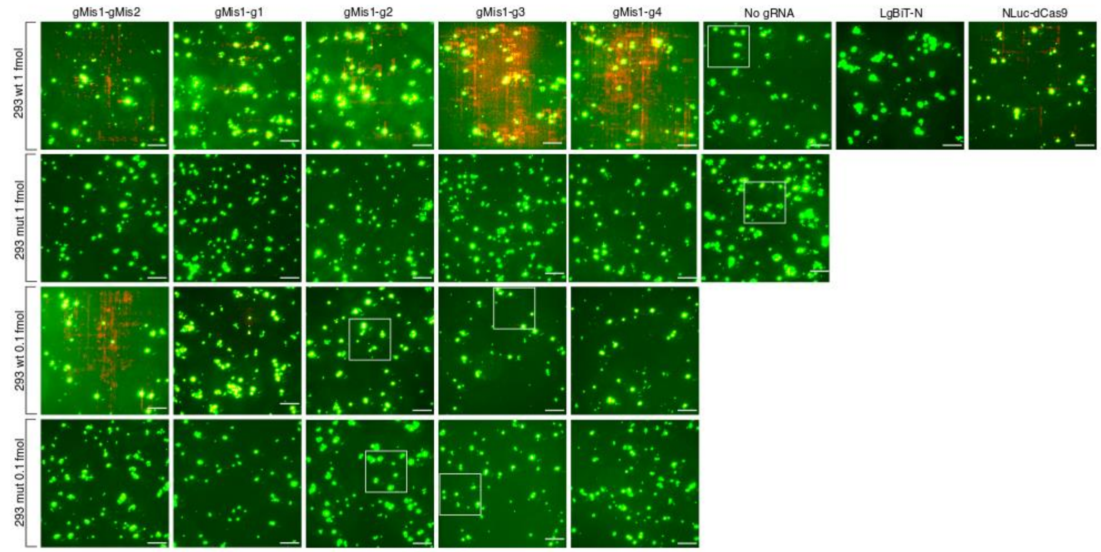

Fig. S6: Panel of images representing merged GFP (fluorescence) and NLuc (luminescence) channels at 10X magnification taken on the Leica DM6000 B upright microscope. Two reduced amounts (1 and 0.1 fmol) of the dCas9-NanoBiT probe were applied to the *PALB2* locus after targeted CRISPR-Cas9 genome editing. Wild-type HEK 293 cells expressing the LgBiT-dCas9 and dCas9-SmBiT constructs and several gRNA pairs are compared to HEK 293 cells homozygous for a G->T missense mutation within an SpCas9 PAM site at the *PALB2* locus expressing the same probe components. Controls with no gRNA transfected, LgBiT-dCas9 alone transfected, and NLuc-dCas9 probe transfected are shown for comparison. Boxes are shown in the above images which depict approximately 437 μM x 437 μM square sections and represent the image sections shown in Fig. 4b. Scale bars=200 μM.

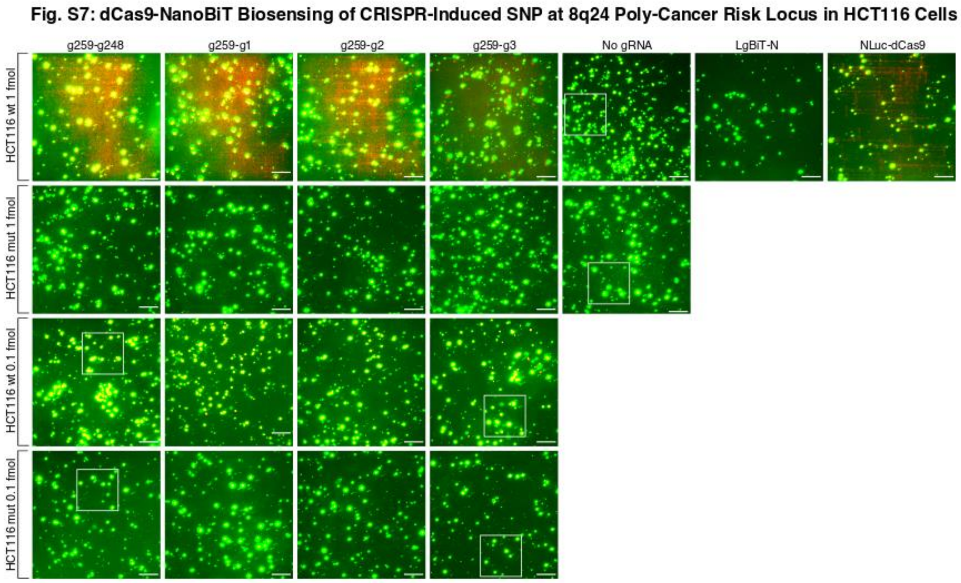

Fig. S7: Panel of images representing merged GFP (fluorescence) and NLuc (luminescence) channels at 10X magnification taken on the Leica DM6000 B upright microscope. Two reduced amounts (1 and 0.1 fmol) of the dCas9-NanoBiT probe were applied to the 8q24 poly-cancer risk locus after targeted CRISPR-Cas9 genome editing. Wild-type HCT116 cells expressing the LgBiT-dCas9 and dCas9-SmBiT constructs and several gRNA pairs are compared to HCT116 cells homozygous for a G->T missense mutation within an SpCas9 PAM site at the 8q24 poly-cancer risk locus expressing the same probe components. Controls with no gRNA transfected, LgBiT-dCas9 alone transfected, and NLuc-dCas9 probe transfected are shown for comparison. Boxes are shown in the above images which depict approximately 437 µM x 437 µM square sections and represent the image sections shown in Fig. 4c. Scale bars=200 µM.

Fig. S8: dCas9-NanoBiT Biosensing at Nonrepetitive *MUC4*

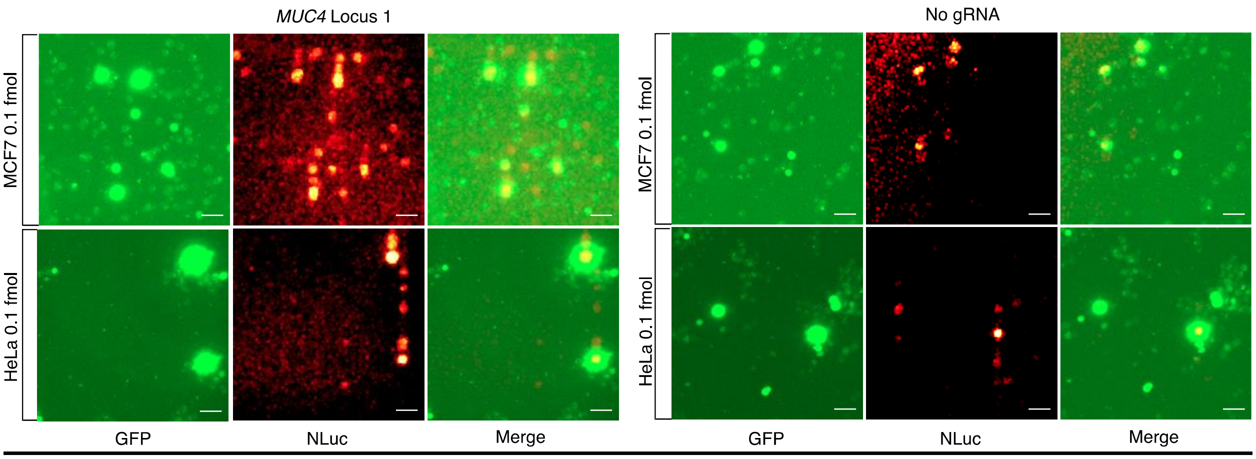

Fig. S8: Panels of images representing individual GFP (fluorescence) and NLuc (luminescence) image channels and corresponding merged images of approximately 437 µM x 437 µM square sections at 10X magnification taken on the Leica DM6000 B upright microscope. Panels depict biosensing images of a single pair of sgRNAs directed to bind at locus 1 of the nonrepetitive region of *MUC4* intron 1 compared to no sgRNA controls for HeLa and MCF7 cells at 0.1 fmol transfected. Scale bars=50 µM.

SUPPLEMENTARY TABLES

Table S1:

Two-way ANOVA	Df	Sum Sq	F value	Pr(>F)
**Fusion Protein Orientation**	3	28557	302.92	<2e-16
**Target DNA Orientation**	32	1498	1.49	0.0494
**FP Orientation:DNA Orientation**	96	6227	2.06	2.94e-06
**Residuals**	264	8296		

Key: **Df:** degrees of freedom for each variable**; Sum sq:** sum of squares**; F value:** the test statistic from the F-test**; Pr(>F):** p-value of the F statistic

Table S2:

Tukey HSD	diff	lwr	upr	p adj
**LC+SN-LC+SC**	-0.9021779	-2.9621672	1.15781142	0.66988987
**LN+SC-LC+SC**	16.3056095	14.2456202	18.3655988	7.57E-14
**LN+SN-LC+SC**	-6.4615939	-8.5215832	-4.4016046	2.33E-13
**LN+SC-LC+SN**	17.2077874	15.1477981	19.2677767	7.57E-14
**LN+SN-LC+SN**	-5.559416	-7.6194053	-3.4994267	1.45E-10
**LN+SN-LN+SC**	-22.767203	-24.827193	-20.707214	7.57E-14

Key: **LN:** LgBiT-dCas9 fusion protein**; LC:** dCas9-LgBiT fusion protein**; SN:** SmBiT-dCas9 fusion protein**; SC:** dCas9-SmBiT fusion protein**; diff:** difference between means of the two groups**; lwr**, **upr:** the lower and the upper end point of the confidence interval at 95% (default)**; p adj:** p-value after adjustment for the multiple comparisons

EXTENDED EXPERIMENTAL PROCEDURES

Experiment 1: Process for Creation of dCas9-NanoBiT Fusion Constructs

gBlocks for initial dCas9-NanoBiT cloning scheme

NLS-HA-LgBiT (Nfus):

TCCATAGAAGACACCGGGACCGATCCAGCCTCCGGACTCTAGAGGATCGAACCCTT
GCCACCATGCCCAAGAAGAAGAGGAAGGTGGGAGGCTCCGGAGGAAGCTACCCAT
ACGATGTCCCAGACTACGCGGGTGGCGGGTCCGGCGGTGGATCCATGGTCTTCACA
CTCGAAGATTTCGTTGGGGACTGGGAACAGACAGCCGCCTACAACCTGGACCAAGT
CCTTGAACAGGGAGGTGTGTCCAGTTTGCTGCAGAATCTCGCCGTGTCCGTAACTCC
GATCCAAAGGATTGTCCGGAGCGGTGAAAATGCCCTGAAGATCGACATCCATGTCA
TCATCCCGTATGAAGGTCTGAGCGCCGACCAAATGGCCCAGATCGAAGAGGTGTTT
AAGGTGGTGTACCCTGTGGATGATCATCACTTTAAGGTGATCCTGCCCTATGGCACA
CTGGTAATCGACGGGGTTACGCCGAACATGCTGAACTATTTCGGACGGCCGTATGAA
GGCATCGCCGTGTTCGACGGCAAAAAGATCACTGTAACAGGGACCCTGTGGAACGG
CAACAAAATTATCGACGAGCGCCTGATCACCCCCGACGGCTCCATGCTGTTCCGAGT
AACCATCAACAGTGGTACCGGAGGGAGTGGTGGAAGCGGCGGTTCTGGTGGCTCAG

NLS-HA-SmBiT (Nfus):

TCCATAGAAGACACCGGGACCGATCCAGCCTCCGGACTCTAGAGGATCGAACCCTT
GCCACCATGCCCAAGAAGAAGAGGAAGGTGGGAGGCTCCGGAGGAAGCTACCCAT
ACGATGTCCCAGACTACGCGGGTGGCGGGTCCGGCGGTGGATCCATGGTGACCGGC
TACCGGCTGTTCGAGGAGATTCTCGGTACCGGAGGGAGTGGTGGAAGCGGCGGTTC
TGGTGGCTCAG

LgBiT-NLS (Cfus):

TAGTGGAGGTTCAGGAGGATCCGGGGGGAGCGGAGGGAGCGCTAGCGTCTTCACAC
TCGAAGATTTCGTTGGGGACTGGGAACAGACAGCCGCCTACAACCTGGACCAAGTC
CTTGAACAGGGAGGTGTGTCCAGTTTGCTGCAGAATCTCGCCGTGTCCGTAACTCCG

ATCCAAAGGATTGTCCGGAGCGGTGAAAATGCCCTGAAGATCGACATCCATGTCAT
CATCCCGTATGAAGGTCTGAGCGCCGACCAAATGGCCCAGATCGAAGAGGTGTTTA
AGGTGGTGTACCCTGTGGATGATCATCACTTTAAGGTGATCCTGCCCTATGGCACAC
TGGTAATCGACGGGGTTACGCCGAACATGCTGAACTATTTCGGACGGCCGTATGAA
GGCATCGCCGTGTTCGACGGCAAAAAGATCACTGTAACAGGGACCCTGTGGAACGG
CAACAAAATTATCGACGAGCGCCTGATCACCCCCGACGGCTCCATGCTGTTCCGAGT
AACCATCAACAGCGGTGGAGGCTCCGGAGGTGGATCTAAAAGGCCGGCGGCCACGA
AAAAGGCCGGTCAGGCAAAAAAGAAAAAGGGTGGTAGTGGAAGCGGAGCGGCCGC
ATGAAAGGGTTCGATCCCTACCGGTTAGTAATGAGT

SmBiT-NLS (Cfus):

TAGTGGAGGTTCAGGAGGATCCGGGGGGAGCGGAGGGAGCGCTAGCGTGACCGGCT
ACCGGCTGTTCGAGGAGATTCTGGGTGGAGGCTCCGGAGGTGGATCTAAAAGGCCG
GCGGCCACGAAAAAGGCCGGTCAGGCAAAAAAGAAAAAGGGTGGTAGTGGAAGCG
GAGCGGCCGCATGAAAGGGTTCGATCCCTACCGGTTAGTAATGAGT

HC91V3 (iCas9V3) vector map:

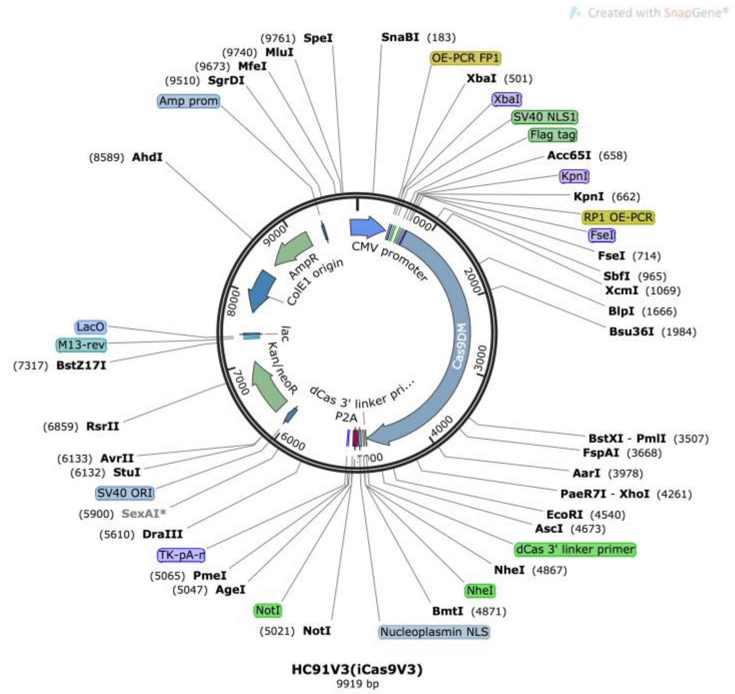

Overlap Extension PCR Primers to create NLuc-dCas9 Fusion Construct:

FP 1 (LgBiT-N gBlock): 5'-TCCATAGAAGACACCGGGAC

RP 1 (LgBiT-N gBlock w/ 5' homology to SmBiT-N gBlock): 5'-
CGAACAGCCGGTAGCCGGTCACACTGTTGATGGTTACTCGGAAC

FP 2 (SmBiT-N gBlock w/ 5' homology to LgBiT-N gBlock): 5'-
GTTCCGAGTAACCATCAACAGTGTGACCGGCTACCGGCTGTTCG

RP 2 (SmBiT-N gBlock): 5'-CTGAGCCACCAGAACCGCCGC

Final verified protein sequences:

NLuc-dCas9:

MPKKKRKVGGSGGSYPYDVPDYAGGGSGGGSMVFTLEDFVGDWEQTAAYNLDQVLE
QGGVSSLLQNLAVSVTPIQRIVRSGENALKIDIHVIIPYEGLSADQMAQIEEVFKVVYPVD
DHHFKVILPYGTLVIDGVTPNMLNYFGRPYEGIAVFDGKKITVTGTLWNGNKIIDERLITP
DGSMLFRVTINSVTGYRLFEEILGTGGSGGSGGSGGSGGSGRPMDKKYSIGLAIGTNSVG
WAVITDEYKVPSKKFKVLGNTDRHSIKKNLIGALLFDSGETAEATRLKRTARRRYTRRK
NRICYLQEIFSNEMAKVDDSFFHRLEESFLVEEDKKHERHPIFGNIVDEVAYHEKYPTIYH
LRKKLVDSTDKADLRLIYLALAHMIKFRGHFLIEGDLNPDNSDVDKLFIQLVQTYNQLFE
ENPINASGVDAKAILSARLSKSRRLENLIAQLPGEKKNGLFGNLIALSLGLTPNFKSNFDL
AEDAKLQLSKDTYDDDLDNLLAQIGDQYADLFLAAKNLSDAILLSDILRVNTEITKAPLS
ASMIKRYDEHHQDLTLLKALVRQQLPEKYKEIFFDQSKNGYAGYIDGGASQEEFYKFIK
PILEKMDGTEELLVKLNREDLLRKQRTFDNGSIPHQIHLGELHAILRRQEDFYPFLKDNR
EKIEKILTFRIPYYVGPLARGNSRFAWMTRKSEETITPWNFEEVVDKGASAQSFIERMTN
FDKNLPNEKVLPKHSLLYEYFTVYNELTKVKYVTEGMRKPAFLSGEQKKAIVDLLFKTN
RKVTVKQLKEDYFKKIECFDSVEISGVEDRFNASLGTYHDLLKIIKDKDFLDNEENEDILE
DIVLTLTLFEDREMIEERLKTYAHLFDDKVMKQLKRRRYTGWGRLSRKLINGIRDKQSG
KTILDFLKSDGFANRNFMQLIHDDSLTFKEDIQKAQVSGQGDSLHEHIANLAGSPAIKKG
ILQTVKVVDELVKVMGRHKPENIVIEMARENQTTQKGQKNSRERMKRIEEGIKELGSQI
LKEHPVENTQLQNEKLYLYYLQNGRDMYVDQELDINRLSDYDVDHIVPQSFLKDDSID
NKVLTRSDKNRGKSDNVPSEEVVKKMKNYWRQLLNAKLITQRKFDNLTKAERGGLSE
LDKAGFIKRQLVETRQITKHVAQILDSRMNTKYDENDKLIREVKVITLKSKLVSDFRKDF

QFYKVREINNYHHAHDAYLNAVVGTALIKKYPKLESEFVYGDYKVYDVRKMIAKSEQE
IGKATAKYFFYSNIMNFFKTEITLANGEIRKRPLIETNGETGEIVWDKGRDFATVRKVLS
MPQVNIVKKTEVQTGGFSKESILPKRNSDKLIARKKDWDPKKYGGFDSPTVAYSVLVV
AKVEKGKSKKLKSVKELLGITIMERSSFEKNPIDFLEAKGYKEVKKDLIIKLPKYSLFELE
NGRKRMLASAGELQKGNELALPSKYVNFLYLASHYEKLKGSPEDNEQKQLFVEQHKH
YLDEIIEQISEFSKRVILADANLDKVLSAYNKHRDKPIREQAENIIHLFTLTNLGAPAAFKY
FDTTIDRKRYTSTKEVLDATLIHQSITGLYETRIDLSQLGGDGGSGGSGGSGGSGGSASG
GGSGGGSKRPAATKKAGQAKKKKGGSGSGATNFSLLKQAGDVEENPGPAAA*

KEY: SV40 NLS, HA epitope, dCas9 (D10A H840A), NLuc, Nucleoplasmin NLS, P2A,
variable length flexible linkers

LgBiT-dCas9:

MPKKKRKVGGSGGSYPYDVPDYAGGGSGGGSMVFTLEDFVGDWEQTAAYNLDQVLE
QGGVSSLLQNLAVSVTPIQRIVRSGENALKIDIHVIIPYEGLSADQMAQIEEVFKVVYPVD
DHHFKVILPYGTLVIDGVTPNMLNYFGRPYEGIAVFDGKKITVTGTLWNGNKIIDERLITP
DGSMLFRVTINSGTGGSGGSGGSGGSGGSGRPMDKKYSIGLAIGTNSVGWAVITDEYKV
PSKKFKVLGNTDRHSIKKNLIGALLFDSGETAEATRLKRTARRRYTRRKNRICYLQEIFS
NEMAKVDDSFFHRLEESFLVEEDKKHERHPIFGNIVDEVAYHEKYPTIYHLRKKLVDST
DKADLRLIYLALAHMIKFRGHFLIEGDLNPDNSDVDKLFIQLVQTYNQLFEENPINASGV
DAKAILSARLSKSRRLENLIAQLPGEKKNGLFGNLIALSLGLTPNFKSNFDLAEDAKLQL
SKDTYDDDLDNLLAQIGDQYADLFLAAKNLSDAILLSDILRVNTEITKAPLSASMIKRYD
EHHQDLTLLKALVRQQLPEKYKEIFFDQSKNGYAGYIDGGASQEEFYKFIKPILEKMDGT
EELLVKLNREDLLRKQRTFDNGSIPHQIHLGELHAILRRQEDFYPFLKDNREKIEKILTFRI
PYYVGPLARGNSRFAWMTRKSEETITPWNFEEVVDKGASAQSFIERMTNFDKNLPNEK
VLPKHSLLYEYFTVYNELTKVKYVTEGMRKPAFLSGEQKKAIVDLLFKTNRKVTVKQL
KEDYFKKIECFDSVEISGVEDRFNASLGTYHDLLKIIKDKDFLDNEENEDILEDIVLTLTLF
EDREMIEERLKTYAHLFDDKVMKQLKRRRYTGWGRLSRKLINGIRDKQSGKTILDFLKS
DGFANRNFMQLIHDDSLTFKEDIQKAQVSGQGDSLHEHIANLAGSPAIKKGILQTVKVV
DELVKVMGRHKPENIVIEMARENQTTQKGQKNSRERMKRIEEGIKELGSQILKEHPVEN

TQLQNEKLYLYYLQNGRDMYVDQELDINRLSDYDVDHIVPQSFLKDDSIDNKVLTRSD
KNRGKSDNVPSEEVVKKMKNYWRQLLNAKLITQRKFDNLTKAERGGLSELDKAGFIKR
QLVETRQITKHVAQILDSRMNTKYDENDKLIREVKVITLKSKLVSDFRKDFQFYKVREIN
NYHHAHDAYLNAVVGTALIKKYPKLESEFVYGDYKVYDVRKMIAKSEQEIGKATAKYF
FYSNIMNFFKTEITLANGEIRKRPLIETNGETGEIVWDKGRDFATVRKVLSMPQVNIVKK
TEVQTGGFSKESILPKRNSDKLIARKKDWDPKKYGGFDSPTVAYSVLVVAKVEKGKSK
KLKSVKELLGITIMERSSFEKNPIDFLEAKGYKEVKKDLIIKLPKYSLFELENGRKRMLAS
AGELQKGNELALPSKYVNFLYLASHYEKLKGSPEDNEQKQLFVEQHKHYLDEIIEQISEF
SKRVILADANLDKVLSAYNKHRDKPIREQAENIIHLFTLTNLGAPAAFKYFDTTIDRKRY
TSTKEVLDATLIHQSITGLYETRIDLSQLGGDGGSGGSGGSGGSGGSASGGGSGGGSKRP
AATKKAGQAKKKKGGSGSGATNFSLLKQAGDVEENPGPAAA*

KEY: SV40 NLS, HA epitope, dCas9 (D10A H840A), LgBiT, Nucleoplasmin NLS, P2A, variable length flexible linkers

SmBiT-dCas9:

MPKKKRKVGGSGGSYPYDVPDYAGGGSGGGSMVTGYRLFEEILGTGGSGGSGGSGGS
GGSGRPMDKKYSIGLAIGTNSVGWAVITDEYKVPSKKFKVLGNTDRHSIKKNLIGALLF
DSGETAEATRLKRTARRRYTRRKNRICYLQEIFSNEMAKVDDSFFHRLEESFLVEEDKK
HERHPIFGNIVDEVAYHEKYPTIYHLRKKLVDSTDKADLRLIYLALAHMIKFRGHFLIEG
DLNPDNSDVDKLFIQLVQTYNQLFEENPINASGVDAKAILSARLSKSRRLENLIAQLPGE
KKNGLFGNLIALSLGLTPNFKSNFDLAEDAKLQLSKDTYDDDLDNLLAQIGDQYADLFL
AAKNLSDAILLSDILRVNTEITKAPLSASMIKRYDEHHQDLTLLKALVRQQLPEKYKEIFF
DQSKNGYAGYIDGGASQEEFYKFIKPILEKMDGTEELLVKLNREDLLRKQRTFDNGSIPH
QIHLGELHAILRRQEDFYPFLKDNREKIEKILTFRIPYYVGPLARGNSRFAWMTRKSEETI
TPWNFEEVVDKGASAQSFIERMTNFDKNLPNEKVLPKHSLLYEYFTVYNELTKVKYVTE
GMRKPAFLSGEQKKAIVDLLFKTNRKVTVKQLKEDYFKKIECFDSVEISGVEDRFNASL
GTYHDLLKIIKDKDFLDNEENEDILEDIVLTLTLFEDREMIEERLKTYAHLFDDKVMKQL
KRRRYTGWGRLSRKLINGIRDKQSGKTILDFLKSDGFANRNFMQLIHDDSLTFKEDIQKA
QVSGQGDSLHEHIANLAGSPAIKKGILQTVKVVDELVKVMGRHKPENIVIEMARENQTT

QKGQKNSRERMKRIEEGIKELGSQILKEHPVENTQLQNEKLYLYYLQNGRDMYVDQEL
DINRLSDYDVDHIVPQSFLKDDSIDNKVLTRSDKNRGKSDNVPSEEVVKKMKNYWRQL
LNAKLITQRKFDNLTKAERGGLSELDKAGFIKRQLVETRQITKHVAQILDSRMNTKYDE
NDKLIREVKVITLKSKLVSDFRKDFQFYKVREINNYHHAHDAYLNAVVGTALIKKYPKL
ESEFVYGDYKVYDVRKMIAKSEQEIGKATAKYFFYSNIMNFFKTEITLANGEIRKRPLIET
NGETGEIVWDKGRDFATVRKVLSMPQVNIVKKTEVQTGGFSKESILPKRNSDKLIARKK
DWDPKKYGGFDSPTVAYSVLVVAKVEKGKSKKLKSVKELLGITIMERSSFEKNPIDFLE
AKGYKEVKKDLIIKLPKYSLFELENGRKRMLASAGELQKGNELALPSKYVNFLYLASHY
EKLKGSPEDNEQKQLFVEQHKHYLDEIIEQISEFSKRVILADANLDKVLSAYNKHRDKPI
REQAENIIHLFTLTNLGAPAAFKYFDTTIDRKRYTSTKEVLDATLIHQSITGLYETRIDLSQ
LGGDGGSGGSGGSGGSGGSASGGGSGGGSKRPAATKKAGQAKKKKGGSGSGATNFSL
LKQAGDVEENPGPAAA*

KEY: SV40 NLS, HA epitope, dCas9 (D10A H840A), SmBiT, Nucleoplasmin NLS, P2A,
variable length flexible linkers

dCas9-LgBiT:

MPKKKRKVGGSGGSDYKDHDGDYKDHDIDYKDDDDKGGGSGGGSGTGGSGGSGGSG
GSGGSGRPMDKKYSIGLAIGTNSVGWAVITDEYKVPSKKFKVLGNTDRHSIKKNLIGAL
LFDSGETAEATRLKRTARRRYTRRKNRICYLQEIFSNEMAKVDDSFFHRLEESFLVEEDK
KHERHPIFGNIVDEVAYHEKYPTIYHLRKKLVDSTDKADLRLIYLALAHMIKFRGHFLIE
GDLNPDNSDVDKLFIQLVQTYNQLFEENPINASGVDAKAILSARLSKSRRLENLIAQLPG
EKKNGLFGNLIALSLGLTPNFKSNFDLAEDAKLQLSKDTYDDDLDNLLAQIGDQYADLF
LAAKNLSDAILLSDILRVNTEITKAPLSASMIKRYDEHHQDLTLLKALVRQQLPEKYKEIF
FDQSKNGYAGYIDGGASQEEFYKFIKPILEKMDGTEELLVKLNREDLLRKQRTFDNGSIP
HQIHLGELHAILRRQEDFYPFLKDNREKIEKILTFRIPYYVGPLARGNSRFAWMTRKSEET
ITPWNFEEVVDKGASAQSFIERMTNFDKNLPNEKVLPKHSLLYEYFTVYNELTKVKYVT
EGMRKPAFLSGEQKKAIVDLLFKTNRKVTVKQLKEDYFKKIECFDSVEISGVEDRFNAS
LGTYHDLLKIIKDKDFLDNEENEDILEDIVLTLTLFEDREMIEERLKTYAHLFDDKVMKQ
LKRRRYTGWGRLSRKLINGIRDKQSGKTILDFLKSDGFANRNFMQLIHDDSLTFKEDIQK

AQVSGQGDSLHEHIANLAGSPAIKKGILQTVKVVDELVKVMGRHKPENIVIEMARENQT
TQKGQKNSRERMKRIEEGIKELGSQILKEHPVENTQLQNEKLYLYYLQNGRDMYVDQE
LDINRLSDYDVDHIVPQSFLKDDSIDNKVLTRSDKNRGKSDNVPSEEVVKKMKNYWRQ
LLNAKLITQRKFDNLTKAERGGLSELDKAGFIKRQLVETRQITKHVAQILDSRMNTKYD
ENDKLIREVKVITLKSKLVSDFRKDFQFYKVREINNYHHAHDAYLNAVVGTALIKKYPK
LESEFVYGDYKVYDVRKMIAKSEQEIGKATAKYFFYSNIMNFFKTEITLANGEIRKRPLIE
TNGETGEIVWDKGRDFATVRKVLSMPQVNIVKKTEVQTGGFSKESILPKRNSDKLIARK
KDWDPKKYGGFDSPTVAYSVLVVAKVEKGKSKKLKSVKELLGITIMERSSFEKNPIDFL
EAKGYKEVKKDLIIKLPKYSLFELENGRKRMLASAGELQKGNELALPSKYVNFLYLASH
YEKLKGSPEDNEQKQLFVEQHKHYLDEIIEQISEFSKRVILADANLDKVLSAYNKHRDKP
IREQAENIIHLFTLTNLGAPAAFKYFDTTIDRKRYTSTKEVLDATLIHQSITGLYETRIDLS
QLGGDGGSGGSGGSGGSGGSASVFTLEDFVGDWEQTAAYNLDQVLEQGGVSSLLQNL
AVSVTPIQRIVRSGENALKIDIHVIIPYEGLSADQMAQIEEVFKVVYPVDDHHFKVILPYG
TLVIDGVTPNMLNYFGRPYEGIAVFDGKKITVTGTLWNGNKIIDERLITPDGSMLFRVTIN
SGGGSGGGSKRPAATKKAGQAKKKKGGSGSGAAA*

KEY: SV40 NLS, 3xFlag epitope, dCas9 (D10A H840A), LgBiT, Nucleoplasmin NLS, variable length flexible linkers

<u>dCas9-SmBiT:</u>

MPKKKRKVGGSGGSDYKDHDGDYKDHDIDYKDDDDKGGGSGGGSGTGGSGGSGGSG
GSGGSGRPMDKKYSIGLAIGTNSVGWAVITDEYKVPSKKFKVLGNTDRHSIKKNLIGAL
LFDSGETAEATRLKRTARRRYTRRKNRICYLQEIFSNEMAKVDDSFFHRLEESFLVEEDK
KHERHPIFGNIVDEVAYHEKYPTIYHLRKKLVDSTDKADLRLIYLALAHMIKFRGHFLIE
GDLNPDNSDVDKLFIQLVQTYNQLFEENPINASGVDAKAILSARLSKSRRLENLIAQLPG
EKKNGLFGNLIALSLGLTPNFKSNFDLAEDAKLQLSKDTYDDDLDNLLAQIGDQYADLF
LAAKNLSDAILLSDILRVNTEITKAPLSASMIKRYDEHHQDLTLLKALVRQQLPEKYKEIF
FDQSKNGYAGYIDGGASQEEFYKFIKPILEKMDGTEELLVKLNREDLLRKQRTFDNGSIP
HQIHLGELHAILRRQEDFYPFLKDNREKIEKILTFRIPYYVGPLARGNSRFAWMTRKSEET
ITPWNFEEVVDKGASAQSFIERMTNFDKNLPNEKVLPKHSLLYEYFTVYNELTKVKYVT

EGMRKPAFLSGEQKKAIVDLLFKTNRKVTVKQLKEDYFKKIECFDSVEISGVEDRFNAS
LGTYHDLLKIIKDKDFLDNEENEDILEDIVLTLTLFEDREMIEERLKTYAHLFDDKVMKQ
LKRRRYTGWGRLSRKLINGIRDKQSGKTILDFLKSDGFANRNFMQLIHDDSLTFKEDIQK
AQVSGQGDSLHEHIANLAGSPAIKKGILQTVKVVDELVKVMGRHKPENIVIEMARENQT
TQKGQKNSRERMKRIEEGIKELGSQILKEHPVENTQLQNEKLYLYYLQNGRDMYVDQE
LDINRLSDYDVDHIVPQSFLKDDSIDNKVLTRSDKNRGKSDNVPSEEVVKKMKNYWRQ
LLNAKLITQRKFDNLTKAERGGLSELDKAGFIKRQLVETRQITKHVAQILDSRMNTKYD
ENDKLIREVKVITLKSKLVSDFRKDFQFYKVREINNYHHAHDAYLNAVVGTALIKKYPK
LESEFVYGDYKVYDVRKMIAKSEQEIGKATAKYFFYSNIMNFFKTEITLANGEIRKRPLIE
TNGETGEIVWDKGRDFATVRKVLSMPQVNIVKKTEVQTGGFSKESILPKRNSDKLIARK
KDWDPKKYGGFDSPTVAYSVLVVAKVEKGKSKKLKSVKELLGITIMERSSFEKNPIDFL
EAKGYKEVKKDLIIKLPKYSLFELENGRKRMLASAGELQKGNELALPSKYVNFLYLASH
YEKLKGSPEDNEQKQLFVEQHKHYLDEIIEQISEFSKRVILADANLDKVLSAYNKHRDKP
IREQAENIIHLFTLTNLGAPAAFKYFDTTIDRKRYTSTKEVLDATLIHQSITGLYETRIDLS
QLGGDGGSGGSGGSGGSGGSASVTGYRLFEEILGGGSGGGSKRPAATKKAGQAKKKKG
GSGSGAAA*

KEY: SV40 NLS, 3xFlag epitope, dCas9 (D10A H840A), SmBiT, Nucleoplasmin NLS, variable length flexible linkers

<u>Experiment 2</u>: Process for Creation of DNA Target Site Plasmids

<u>gBlock 1 Sequence with tandem A and B target sites:</u>

GGGTTTGCTGCTCATCTATACTTTCACAATCTTGAGCTGCAGGGCAAAGAGCTCCCTACGCATGCGTCCCAGGCAGCGTGTATAGT
GAAAAGGAACCCGGGGATGGAGGAAGGGACATAGGGAGATGGCTCAGGTTTGTCGCGCGGTATGTAGCATGGCCCGGGAAGTACAG
TAGAGCTCCCTACGCATGCGTCCCAGGTGCTACTTACATATTCTCCCGGGTAAATTAATTCTTATGAGATGGCTCAGGTTTGTC GC
GCGGCTAGTAGCCCGGGGCATTGTGCTCCCTACGCATGCGTCCCAGGATCTAATCATATCCCGGGATGAAGGTCTATGATGGCTCA
GGTTTGTCGCGCGGTATGCTGAATAATTGAGCCCGGGATAGTGAAATTTATGATGCTCCCTACGCATGCGTCCCAGGTGCTTTTCC
CGGGTGCACAAGATGGCTCAGGTTTGTCGCGCGGAATATAATAATATGTAGATGGTCCCGGGTAGGTTGTTATACATTTACT GAGC
TCCCTACGCATGCGTCCCAGGTTTGTAGAAGGCTAGGGGAACAGGTTAGTTTGAGGGAATTCTAATGGATCCTTCTATGGG

1. **<u>PCR and OE-PCR on gBlock 1 to generate spacers from 6 bp to 50 bp. Red indicates mispriming), Green indicates Target Site A, Blue indicates Target Site B</u>**

<u>6 bp spacer:</u>

FP1: 5’ – CTTGAGCTGCAGGGCAAA (Tm 68) (Taq 58)

RP1: 5’ – CGACAAACCTGAGCCATCTCCCTGCCTGGGACGCATGC (Tm 72) (Taq 63)

FP2: 5’ – GCATGCGTCCCAGGCAGGGAGATGGCTCAGGTTTGTCG (Tm 70) (Taq 61)

RP2: 5’ – TTCCCGGGCCATGCTACA (Tm 72) (Taq 62)

-FP1 and RP1 generate 64bp product, FP2 and RP2 generate 64bp product

-FP1 and RP2 with round 1 products as templates generate 92bp product

Final seq: 5’ –
CTTGAGCTGCAGGGCAAAGAGCTCCCTACGCATGCGTCCCAGGCAGGGAGATGGCTCAGGTTTGTCGCGCGGTATGTAGCATGGCCCGGGAA

10 bp spacer:

FP1: 5’ – CTTGAGCTGCAGGGCAAA (Tm 68) (Taq 58)

RP1: 5’ – CAAACCTGAGCCATCTCCCTCGCTGCCTGGGACGCAT (Tm 74) (Taq 65)

FP2: 5’ – ATGCGTCCCAGGCAGCGAGGGAGATGGCTCAGGTTTG (Tm 68) (Taq 59)

RP2: 5’ – TTCCCGGGCCATGCTACA (Tm 72) (Taq 62)

-FP1 and RP1 generate 66bp product, FP2 and RP2 generate 66bp product

-FP1 and RP2 with round 1 products as templates generate 96bp product

Final seq: 5’ –
CTTGAGCTGCAGGGCAAAGAGCTCCCTACGCATGCGTCCCAGGCAGCGAGGGAGATGGCTCAGGTTTGTCGCGCGGTATGTAGCATGGCCCGGGAA

15 bp spacer:

FP1: 5’ – CTTGAGCTGCAGGGCAAA (Tm 68) (Taq 58)

RP1: 5’ – CAAACCTGAGCCATCTCCCTATACACGCTGCCTGGGACG (Tm 72) (Taq 65)

FP2: 5’ – CGTCCCAGGCAGCGTGTATAGGGAGATGGCTCAGGTTTG (Tm 67) (Taq 59)

RP2: 5’ – TTCCCGGGCCATGCTACA (Tm 72) (Taq 62)

-FP1 and RP1 generate 69bp product, FP2 and RP2 generate 68bp product

-FP1 and RP2 with round 1 products as templates generate 101bp product

Final seq: 5’ –
CTTGAGCTGCAGGGCAAAGAGCTCCCTACGCATGCGTCCCAGGCAGCGTGTATAGGGAGATGGCTCAGGTTTGTCGCGCGGTATGTAGCATGGCCCGGGAA

20 bp spacer:

FP1: 5’ – GGGATAGTGAAATTTATGAT (Tm 54) (Taq 45)

RP1: 5’ – GGGACCATCTACATATTATTATATT (Tm 57) (Taq 48)

-FP1 and RP1 produce 111bp product

Final seq: 5’ – GGGATAGTGAAATTTATGATGCTCCCTACGCATGCGTCCCAGGTGCTTTTCCCGGGTGCACAAGATGGCTCAGGTTTGTCGCGCGGAATATAATAATATGTAGATGGTCCC

25 bp spacer:

FP1: 5’ – CTTGAGCTGCAGGGCAAA (Tm 68) (Taq 58)

RP1: 5’ – GAGCCATCTCCCTATGTCCCTCTATACACGCTGCCTGGG (Tm 64) (Taq 58)

FP2: 5’ – CCCAGGCAGCGTGTATAGAGGGACATAGGGAGATGGCTC (Tm 68) (Taq 61)

RP2: 5’ – TTCCCGGGCCATGCTACA (Tm 72) (Taq 62)

-FP1 and RP1 generate 73bp product, FP2 and RP2 generate 74bp product

-FP1 and RP2 with round 1 products as templates generate 111bp product

Final seq: 5’ – CTTGAGCTGCAGGGCAAAGAGCTCCCTACGCATGCGTCCCAGGCAGCGTGTATAGAGGGACATAGGGAGATGGCTCAGGTTTGTCGCGCGGTATGTAGCATGGCCCGGGAA

30 bp spacer:

FP1: 5’ – CTAGTAGCCCGGGGCATT (Tm 66) (Taq 60)

RP1: 5’ – GGGCTCAATTATTCAGCATA (Tm 61) (Taq 51)

-FP1 and RP1 produce 116bp product

Final seq: 5’ – CTAGTAGCCCGGGGCATTGTGCTCCCTACGCATGCGTCCCAGGATCTAATCATATCCCGGGATGAAGGTCTATGATGGCTCAGGTTTGTCGCGCGGTATGCTGAATAATTGAGCCC

35 bp spacer:

FP1: 5’ – CTTGAGCTGCAGGGCAAA (Tm 68) (Taq 58)

RP1: 5’ – GCCATCTCCCTATGTCCCTTCCTTTTTCACTATACACGCTGCCTG (Tm 66) (Taq 57)

FP2: 5’ – CAGGCAGCGTGTATAGTGAAAAAGGAAGGGACATAGGGAGATGGC (Tm 71) (Taq 63)

RP2: 5’ – TTCCCGGGCCATGCTACA (Tm 72) (Taq 62)

-FP1 and RP1 generate 78bp product, FP2 and RP2 generate 73bp product

-FP1 and RP2 with round 1 products as templates generate 121bp product

Final seq: 5’ –
CTTGAGCTGCAGGGCAAAGAGCTCCCTACGCATGCGTCCCAGGCAGCGTGTATAGTGAAAAAGGAAGGGACATAGGGAGATGGCTC
AGGTTTGTCGCGCGGTATGTAGCATGGCCCGGGAA

40 bp spacer:

FP1: 5’ – GGCCCGGGAAGTACAGTAGA (Tm 67) (Taq 61)

RP1: 5’ – ACAATGCCCCGGGCTACTAG (Tm 69) (Taq 62)

-FP1 and RP1 produce 126bp product

Final seq: 5’ –
GGCCCGGGAAGTACAGTAGAGCTCCCTACGCATGCGTCCCAGGTGCTACTTACATATTCTCCCGGGTAAATTAATTCTTATGAGAT
GGCTCAGGTTTGTCGCGCGGCTAGTAGCCCGGGGCATTGT

50 bp spacer:

FP1: 5’ – CTTGAGCTGCAGGGCAAA (Tm 68) (Taq 58)

RP1: 5’ – TTCCCGGGCCATGCTACA (Tm 72) (Taq 62)

-FP1 and RP1 produce 136bp product

Final seq: 5’ –
CTTGAGCTGCAGGGCAAAGAGCTCCCTACGCATGCGTCCCAGGCAGCGTGTATAGTGAAAAGGAACCCGGGGATGGAGGA
AGGGACATAGGGAGATGGCTCAGGTTTGTCGCGCGGTATGTAGCATGGCCCGGGAA

gBlock 2 with inverted A and B target sites:

GGGTTTGCTGCTCATCTATACTTTCACAATCTTGAGCTGCAGGGCAAAGACTACAATGGGATTAATAAATTGTACTCTAA
AGGATATTGAAAACTTGTGAGCTCCCTACGCATGCGTCCCAGGCAGCGTGTATAGTGAAAAGGAACCCGGGGATGGAGGA
AGGGACATAGGGACCGCGCGACAAACCTGAGCCATCTATGTAGCATGGCCCGGGAAGTACAGTAGAGCTCCCTACGCATG
CGTCCCAGGTGCTACTTACATATTCTCCCGGGTAAATTAATTCTTATGACCGCGCGACAAACCTGAGCCATCCTAGTAGC
CCGGGGCATTGTGCTCCCTACGCATGCGTCCCAGGATCTAATCATATCCCGGGATGAAGGTCTATCCGCGCGACAAACCT
GAGCCATCTATGCTGAATAATTGAGCCCGGGATAGTGAAATTTATGATGCTCCCTACGCATGCGTCCCAGGTGCTTTTCC
CGGGTGCACAACCGCGCGACAAACCTGAGCCATCAATATAATAATATGTAGATGGTCCCGGGTAGGTTGTTATACATTTA
CTGAGCTCCCTACGCATGCGTCCCAGGTTTGTAGAAGGCTAGGGGAACAGGTTAGTTTGAGGGAATTCTAATGGATCCTT
CTATGGG

2. **PCR and OE-PCR on gBlock 2 to generate spacers from 6 bp to 50 bp. Red indicates mispriming), Green indicates Target Site A, Blue indicates Target Site B**

6 bp spacer:

FP1: 5’ – ACTCTAAAGGATATTGAAAACTTGTGA (Tm 63) (Taq 53)

RP1: 5’ – AGGTTTGTCGCGCGGTCCCTGCCTGGGACGCATG (Tm 69) (Taq 60)

FP2: 5’ – CATGCGTCCCAGGCAGGGACCGCGCGACAAACCT (Tm 73) (Taq 64)

RP2: 5’ – ACTGTACTTCCCGGGCCA (Tm 68) (Taq 61)

-FP1 and RP1 generate 70bp product, FP2 and RP2 generate 70bp product

-FP1 and RP2 with round 1 products as templates generate 106bp product

Final seq: 5’ –

ACTCTAAAGGATATTGAAAACTTGTGAGCTCCCTACGCATGCGTCCCAGGCAGGGACCGCGCGACAAACCTGAGCCATCTATGTAG
CATGGCCCGGGAAGTACAGT

10 bp spacer:

FP1: 5’ – ACTCTAAAGGATATTGAAAACTTGTGA (Tm 63) (Taq 53)

RP1: 5’ – GTTTGTCGCGCGGTCCCTCGCTGCCTGGGACGCAT (Tm 74) (Taq 65)

FP2: 5’ – ATGCGTCCCAGGCAGCGAGGGACCGCGCGACAAAC (Tm 73) (Taq 64)

RP2: 5’ – ACTGTACTTCCCGGGCCA (Tm 68) (Taq 61)

-FP1 and RP1 generate 73bp product, FP2 and RP2 generate 72bp product

-FP1 and RP2 with round 1 products as templates generate 110bp product

Final seq: 5’ –
ACTCTAAAGGATATTGAAAACTTGTGAGCTCCCTACGCATGCGTCCCAGGCAGCGAGGGACCGCGCGACAAACCTGAGCCATCTAT
GTAGCATGGCCCGGGAAGTACAGT

15 bp spacer:

FP1: 5’ – ACTCTAAAGGATATTGAAAACTTGTGA (Tm 63) (Taq 53)

RP1: 5’ – GTTTGTCGCGCGGTCCCTATACACGCTGCCTGGGAC (Tm 66) (Taq 60)

FP2: 5’ – GTCCCAGGCAGCGTGTATAGGGACCGCGCGACAAAC (Tm 73) (Taq 64)

RP2: 5’ – ACTGTACTTCCCGGGCCA (Tm 68) (Taq 61)

-FP1 and RP1 generate 78bp product, FP2 and RP2 generate 73bp product

-FP1 and RP2 with round 1 products as templates generate 115bp product

Final seq: 5’ –
ACTCTAAAGGATATTGAAAACTTGTGAGCTCCCTACGCATGCGTCCCAGGCAGCGTGTATAGGGACCGCGCGACAAACCTGAGCCA
TCTATGTAGCATGGCCCGGGAAGTACAGT

20 bp spacer:

FP1: 5' - GGGATAGTGAAATTTATGAT (Tm 54) (Taq 45)

RP1: 5' - GGGACCATCTACATATTATTATATT (Tm 57) (Taq 48)

-FP1 and RP1 produce 111bp product

Final seq: 5' -
GGGATAGTGAAATTTATGATGCTCCCTACGCATGCGTCCCAGGTGCTTTTCCCGGGTGCACAACCGCGCGACAAACCTGAGCCATC
AATATAATAATATGTAGATGGTCCC

25 bp spacer:

FP1: 5' - ACTCTAAAGGATATTGAAAACTTGTGA (Tm 63) (Taq 53)

RP1: 5' - GCGGTCCCTATGTCCCTCTATACACGCTGCCTGG (Tm 60) (Taq 55)

FP2: 5' - CCAGGCAGCGTGTATAGAGGGACATAGGGACCGC (Tm 65) (Taq 59)

RP2: 5' - ACTGTACTTCCCGGGCCA (Tm 68) (Taq 61)

-FP1 and RP1 generate 79bp product, FP2 and RP2 generate 80bp product

-FP1 and RP2 with round 1 products as templates generate 125bp product

Final seq: 5' -
ACTCTAAAGGATATTGAAAACTTGTGAGCTCCCTACGCATGCGTCCCAGGCAGCGTGTATAGAGGGACATAGGGACCGCGCGACAA
ACCTGAGCCATCTATGTAGCATGGCCCGGGAAGTACAGT

30 bp spacer:

FP1: 5' - CTAGTAGCCCGGGGCATT (Tm 66) (Taq 60)

RP1: 5' - GGGCTCAATTATTCAGCATA (Tm 61) (Taq 51)

-FP1 and RP1 produce 116bp product

Final seq: 5' -
CTAGTAGCCCGGGGCATTGTGCTCCCTACGCATGCGTCCCAGGATCTAATCATATCCCGGGATGAAGGTCTATCCGCGCGACAAAC
CTGAGCCATCTATGCTGAATAATTGAGCCC

35 bp spacer:

FP1: 5' - ACTCTAAAGGATATTGAAAACTTGTGA (Tm 63) (Taq 53)

RP1: 5' - GGTCCCTATGTCCCTTCCTTTTTCACTATACACGCTGCCT (Tm 63) (Taq 56)

FP2: 5' - AGGCAGCGTGTATAGTGAAAAAGGAAGGGACATAGGGACC (Tm 64) (Taq 58)

RP2: 5' - ACTGTACTTCCCGGGCCA (Tm 68) (Taq 61)

-FP1 and RP1 generate 87bp product, FP2 and RP2 generate 88bp product

-FP1 and RP2 with round 1 products as templates generate 135bp product

Final seq: 5' –
ACTCTAAAGGATATTGAAAACTTGTGAGCTCCCTACGCATGCGTCCCAGGCAGCGTGTATAGTGAAAAAGGAAGGGACATAGGGAC
CGCGCGACAAACCTGAGCCATCTATGTAGCATGGCCCGGGAAGTACAGT

40 bp spacer:

FP1: 5' – GGCCCGGGAAGTACAGTAGA (Tm 67) (Taq 61)

RP1: 5' – ACAATGCCCCGGGCTACTAG (Tm 69) (Taq 62)

-FP1 and RP1 produce 126bp product

Final seq: 5' –
GGCCCGGGAAGTACAGTAGAGCTCCCTACGCATGCGTCCCAGGTGCTACTTACATATTCTCCCGGGTAAATTAATTCTTATGACCG
CGCGACAAACCTGAGCCATCCTAGTAGCCCGGGGCATTGT

50 bp spacer:

FP1: 5' – ACTCTAAAGGATATTGAAAACTTGTGA (Tm 63) (Taq 53)

RP1: 5' – ACTGTACTTCCCGGGCCA (Tm 68) (Taq 61)

-FP1 and RP1 produce 150bp product

Final seq: 5' –
ACTCTAAAGGATATTGAAAACTTGTGAGCTCCCTACGCATGCGTCCCAGGCAGCGTGTATAGTGAAAAGGAACCCGGGGA
TGGAGGAAGGGACATAGGGACCGCGCGACAAACCTGAGCCATCTATGTAGCATGGCCCGGGAAGTACAGT

Everted A & B Sites – Target B Rev followed by Target A Fwd

3. **PCR and OE-PCR on gBlock 2 to generate spacers from 6 bp to 50 bp. Red indicates mispriming), Green indicates Target Site A, Blue indicates Target Site B**

6 bp spacer:

FP1: 5' – CCGGGTAAATTAATTCTTATGA (Tm 60) (Taq 49)

RP1: 5' – GCATGCGTAGGGAGCACATAGGATGGCTCAGGTTTG (Tm 61) (Taq 53)

FP2: 5' – CAAACCTGAGCCATCCTATGTGCTCCCTACGCATGC (Tm 69) (Taq 61)

RP2: 5' – ATCTAATCATATCCCGGGATGA (Tm 65) (Taq 54)

-FP1 and RP1 generate 66bp product, FP2 and RP2 generate 66bp product

-FP1 and RP2 with round 1 products as templates generate 96bp product

Final seq: 5' –

CCGGGTAAATTAATTCTTATGACCGCGCGACAAACCTGAGCCATCCTATGTGCTCCCTACGCATGCGTCCCAGGATCTAATCATAT
CCCGGGATGA

10 bp spacer:

FP1: 5' - CCGGGTAAATTAATTCTTATGA (Tm 60) (Taq 49)

RP1: 5' - ATGCGTAGGGAGCACAATACTAGGATGGCTCAGGTT (Tm 59) (Taq 54)

FP2: 5' - AACCTGAGCCATCCTAGTATTGTGCTCCCTACGCAT (Tm 63) (Taq 56)

RP2: 5' - ATCTAATCATATCCCGGGATGA (Tm 65) (Taq 54)

-FP1 and RP1 generate 68bp product, FP2 and RP2 generate 68bp product

-FP1 and RP2 with round 1 products as templates generate 100bp product

Final seq: 5' -
CCGGGTAAATTAATTCTTATGACCGCGCGACAAACCTGAGCCATCCTAGTATTGTGCTCCCTACGCATGCGTCCCAGGATCTAATC
ATATCCCGGGATGA

15 bp spacer:

FP1: 5' - CCGGGTAAATTAATTCTTATGA (Tm 60) (Taq 49)

RP1: 5' - CGTAGGGAGCACAATGCCCTACTAGGATGGCTCAGG (Tm 57) (Taq 53)

FP2: 5' - CCTGAGCCATCCTAGTAGGGCATTGTGCTCCCTACG (Tm 67) (Taq 59)

RP2: 5' - ATCTAATCATATCCCGGGATGA (Tm 65) (Taq 54)

-FP1 and RP1 generate 70bp product, FP2 and RP2 generate 71bp product

-FP1 and RP2 with round 1 products as templates generate 105bp product

Final seq: 5' -

CCGGGTAAATTAATTCTTATGACCGCGCGACAAACCTGAGCCATCCTAGTAGGGCATTGTGCTCCCTACGCATGCGTCCCAGGATC
TAATCATATCCCGGGATGA

20 bp spacer:

FP1: 5' - CCGGGTAAATTAATTCTTATGA (Tm 60) (Taq 49)

RP1: 5' - ATCTAATCATATCCCGGGATGA (Tm 65) (Taq 54)

-FP1 and RP1 produce 110bp product

Final seq: 5' -
CCGGGTAAATTAATTCTTATGACCGCGCGACAAACCTGAGCCATCCTAGTAGCCCGGGGCATTGTGCTCCCTACGCATGCGTCCCA
GGATCTAATCATATCCCGGGATGA

25 bp spacer:

FP1: 5′ - TTCTCCCGGGTAAATTAATTCTTATGA (Tm 67) (Taq 55)

RP1: 5′ - GGGAGCACAATGCCCCGGCCCGGGCTACTAGGATGG (Tm 67) (Taq 60)

FP2: 5′ - CCATCCTAGTAGCCCGGGCCGGGGCATTGTGCTCCC (Tm 76) (Taq 66)

RP2: 5′ - GACCTTCATCCCGGGATATGATTAGAT (Tm 71) (Taq 60)

-FP1 and RP1 generate 81bp product, FP2 and RP2 generate 80bp product

-FP1 and RP2 with round 1 products as templates generate 125bp product

Final seq: 5′ -
TTCTCCCGGGTAAATTAATTCTTATGACCGCGCGACAAACCTGAGCCATCCTAGTAGCCCGGGCCGGGGCATTGTGCTCCCTACGC
ATGCGTCCCAGGATCTAATCATATCCCGGGATGAAGGTC

30 bp spacer:

FP1: 5′ - GATGGAGGAAGGGACATAGG (Tm 65) (Taq 57)

RP1: 5′ - CCGGGAGAATATGTAAGTAGCA (Tm 64) (Taq 56)

-FP1 and RP1 produce 120bp product

Final seq: 5′ -
GATGGAGGAAGGGACATAGGGACCGCGCGACAAACCTGAGCCATCTATGTAGCATGGCCCGGGAAGTACAGTAGAGCTCCCTACGC
ATGCGTCCCAGGTGCTACTTACATATTCTCCCGG

35 bp spacer:

FP1: 5′ - GATGGAGGAAGGGACATAGG (Tm 65) (Taq 57)

RP1: 5′ - AGCTCTACTGTACTTCCCGGGCCGGGCCATGCTACATAGAT (Tm 68) (Taq 59)

FP2: 5′ - ATCTATGTAGCATGGCCCGGCCCGGGAAGTACAGTAGAGCT (Tm 66) (Taq 61)

RP2: 5′ - CCGGGAGAATATGTAAGTAGCA (Tm 64) (Taq 56)

-FP1 and RP1 generate 83bp product, FP2 and RP2 generate 83bp product

-FP1 and RP2 with round 1 products as templates generate 125bp product

Final seq: 5′ -
GATGGAGGAAGGGACATAGGGACCGCGCGACAAACCTGAGCCATCTATGTAGCATGGCCCGGCCCGGGAAGTACAGTAGAGCTCCC
TACGCATGCGTCCCAGGTGCTACTTACATATTCTCCCGG

40 bp spacer:

FP1: 5′ - ATCCCGGGATGAAGGTCTAT (Tm 66) (Taq 57)

RP1: 5' – TTGTGCACCCGGGAAAA (Tm 69) (Taq 57)

-FP1 and RP1 produce 126bp product

Final seq: 5' –
ATCCCGGGATGAAGGTCTATCCGCGCGACAAACCTGAGCCATCTATGCTGAATAATTGAGCCCGGGATAGTGAAATTTATGATGCTCCCTACGCATGCGTCCCAGGTGCTTTTCCCGGGTGCACAA

50 bp spacer:

FP1: 5' – TTTTCCCGGGTGCACAAC (Tm 69) (Taq 59)

RP1: 5' – GTTCCCCTAGCCTTCTACAAACC (Tm 67) (Taq 60)

-FP1 and RP1 produce 134bp product

Final seq: 5' –
TTTTCCCGGGTGCACAACCGCGCGACAAACCTGAGCCATCAATATAATAATATGTAGATGGTCCCGGGTAGGTTGTTATACATTTACTGAGCTCCCTACGCATGCGTCCCAGGTTTGTAGAAGGCTAGGGGAAC

Experiment 3: Process for Creation of gRNAs

JL gRNAs oligos for annealing to create JL1 and JL2 gRNAs

JL gRNA1

gRNA1 = GCTCCCTACGCATGCGTCCC
DNA target site 1/A (fwd) = GCTCCCTACGCATGCGTCCC**AGG** (no 100% match within hg38 and lowest off-targets (CFD score, CRISPRscan))

JL gRNA2

gRNA2 = GATGGCTCAGGTTTGTCGCG
DNA target site 2/B (fwd) = GATGGCTCAGGTTTGTCGCG**CGG** (no 100% match within hg38 and second lowest off-targets (CFD score, CRISPRscan))

Insert_F: TTTCTTGGCTTTATATATCTTGTGGAAAGGACGAAACACC***G****NNNNNNNNNNNNNNNNNNNN*

Insert_R: GACTAGCCTTATTTTAACTTGCTATTTCTAGCTCTAAAAC*NNNNNNNNNNNNNNNNNNNN****C***

JL gRNA1 F

TTTCTTGGCTTTATATATCTTGTGGAAAGGACGAAACACC***G****CTCCCTACGCATGCGTCCC*

JL gRNA1 R

GACTAGCCTTATTTTAACTTGCTATTTCTAGCTCTAAAAC*GGGACGCATGCGTAGGGAG****C***

JL gRNA2 F

TTTCTTGGCTTTATATATCTTGTGGAAAGGACGAAACACC***G****ATGGCTCAGGTTTGTCGCG*

JL gRNA2 R
GACTAGCCTTATTTTAACTTGCTATTTCTAGCTCTAAAAC*CGCGACAAACCTGAGCCAT***C***

Experiment 4: Process for Creation of gRNAs for MUC4 DNA Biosensing

Repetitive region in exon 2:

MUC4 repetitive DNA region—48 bp repeat:

5′-GCCACCCCTCTTCCTGTCACCGACACTTCCTCAGCATCCACAGGTCAC~GCC-3′
3′-CGGT***G****GGG***AGAA*GGA*CAGTGGCTGTGAAGGAGTCGTAGGTGTCCAGTG~CGG-5′

sgMUC4-E3(F+E): GGCGTGACCTGTGGATGCTGAGG

MUC4 gRNA tgt 1: GACACTTCCTCAGCATCCACAGG

-Everted overlapping, PAMs 10 bp apart
-CFD:110.89

MUC4 gRNA tgt 2: GGTGGATGCTGAGGAAGTGTCGG

-Tandem overlapping, PAMs 6 bp apart
-CFD:163.22

MUC4 gRNA tgt 3: GGTGAGGAAGTGTCGGTGAC*AGG*

-Tandem overlapping, PAMs 13 bp apart
-CFD:70.68

MUC4 gRNA tgt 4: GAAGTGTCGGTGACAGGAAG**A**GG

-Tandem overlapping by 1 bp, PAMs 19 bp apart
-CFD:118.16

MUC4 gRNA tgt 5: GGTGTCGGTGACAGGAAGAG*GG****G***

-Tandem 1 bp
-CFD:122.54

MUC4 gRNA tgt 6: GGCGGTGACAGGAAGAGGGGTGG

-Tandem 4 bp

-CFD:227.72

Selected gRNAs 1-4 for experiments

Non-repetitive region in intron 1:

MUC4 non-repetitive DNA region with Cas9 target sites:

ATGAAGGGGGCACGCTGGAGGAGGGTCCCCTGGGTGTCCCTGAGCTGCCTGTGTCTCTGCCTCC
TTCCGCATGTGGTCCCAGGTAAGTGATGGAGACAGCAGATGAGGCTGGCTGCGGGGAGCACTTG
GGGGAGGTGGGAGCTGTCAGAGAAAGAGGTCCGGGGAGACAGAGAGAGAGAGAGAGAGAATAGG
GGAAAGGGAGACAGCGAAGAGGAAGAGAAGGGAGAGAAAAAGAGGGAGAGGGAAAGGAGAAAGA
GATGAATGGGACAACATGGGGGGAAGGTGGAGAGAGACCCAGAGAGGGAAAGAAGAGGAAGAGA
AGAGGGAGAGAGAAAGAAGAGTGGAGGCCGTGCGCGG**TGG**CTCATGCCTGTAATCCCAGCACTT
TCGGAGG**CCA**AGGCAGGAGATCACCTGAGGTCAGGAGTTCGAGACCAGCCTGGCCGACATGGTG
AAA**CCC**CGTCTCTACTAAATATACAAAAATTAGCCGGTCGTGGTGGG**CCC**CACCTGTAATTCCA
GCTACTCAGGAGTCTGAGGCAGGAGAATCACTTGAA**CCT**GGGAGGTGGAGGTTGCAGTGAGCCA
AGATCGCGCCACTGCACTCCAG**CCT**GGGAGAGAGAGCGAGACTCTGTCTCAAAATAAATAAATA
AATAAATAAATAAATAAATAAATAAATAAATATAAAATAAAATAAAAAATAAGAAGAGGAGAAA
AGTGGGGAAG**AGG**AGGCATGAACTGGCAGATACGGGACAAGATCTGAGGGGAAAGACAGAGGGA
GAATGCTCGAAAGAGAGAGAAAAGAGAACAGAGGGCCA*GAGAGCAGCC**CGG**CGATGTCTGGAAG*
GATCCATGGTGAGAG**CCC**AGGCTTACTCGCAGAGAGAAAGACAGGCAGAGCCAGAGCAAGAGGA
ACAGAGTCAAGGAGAAAGATGTACACCCTTGTGTACAGAGCT**GGG**GGTAGAGGGGATGCCAGGA
AAGCTGGGTGATGGAGACGGAAGAAAACTCATGTAAAGCTGCA**GGG**TGAGAGGACGAGACAGGT
GAGACGCAGACAAACTGAGGACCCTGGGAATGGAGAGAGGAGAAGATCGGGAGACAGCAGCAAG
CAAGGGAAGCGACAAGG**AGG**AGAGGGGCAGGCCGGCCGGGAGGGTGGTGCGGAGGAGGCGGCCA
GGGCGCAGA**GGG**CCGGGAGGTGCTGGCCGTGGGCTTCTTA**CCT**CTGAGCTCGGGTTTAAAAGCC
TCCATTTGGGTCACGGCCTTGCCTGGGGCTCGTAGCCCCGGCATTGGCCTT**GGG**CTCCTCCGTG
TACAGAGCTGGGAGGGGAGGGATGCCAGGCCTGTGGGAGATGTTCCCTC**GGG**GGCCCCCGTCCT
CTTCCCCACACTTT**CCA**GGCTGTCCCTCTGGCTTCAGGACCAAGTTTTATTCTGTGTTTCTGGG
TGTCTGAGTCTTTGGGGGAGAGTCT**GGG**GTCCAGAGTTCAAGCTGGGGTTAGAGTCTCAGCTCC
TGCCCTGCCTCTCAGC**AGG**CTAAGAACAGTCGCCGAGGGAAAATATTTCTTGGGCGCATATTTG
AGGAGCTTCCT**GGG**AGTGAGTCAGAAGGCGAGTGCCGTTTAAAGGCTGCAAGAGAAGCCATGC**T**
GGTGAAGCGGACCCTTCCACCTCGGGATGTTTCAGGACTAGGCTGA**GGG**CAAAGGAAACTGCCA

CCACCT**CCC**TACACCTCCCCACCCTCCAGCACCCCCACCCCACCCTGGCCACACAAC**CCC**GCTC

CAGTGCTCATCCCACCGTGAGGACGTGGAGGCCGGAAGGAGCCGCCACACGGCCCTGCCCTGCA

GATGTGGTTGA**AGG**AGTCTCCACGGGAATCATGACTCCCAGAGCGAGGCTGGGGCTTGGGGCGC

C**GGG**GAGGCAGCTTGGATTTAGGAGCCCCAGGGCCAAGTCTTTGCCGTGAACTGTTC**TGG**CCCC

TGTGACCAGGCCCTGC**CCC**GTGTCTCCCCAGGGCCCCGGTCCCCTGTGTAAAAAGCAGTGGTGA

ACGGTTGGACCTCCTGACGCCCAAGTTCTTGAGTTTCCAAATCTGTGATTTAAAGCTGAGCCCA

AATGTGCTGGGTACCAGCTGGACACTCAGCTCCATG**TGG**AGCCAGGAAGTGGGGTCTGTGGAGA

GGAGCGCAGAGGGGCAAGACCT**GGG**GTGGGCGTGGAAAAGCACGGGGGCGTGACCCGGAGAAGG

AGTGAAGGACTGT**TGG**TGTGCAAGGGCGTCTCCATGACGA**CCC**GAAGAAGCTAGGCATGTCGTG

GAGCGCT*GAGTCCTTTGCGTCGCTAAGGGG*ACCAAGTGGAGCTGGGCCAGGAGAGGAGA**TGG**TC

GTGGCTGGGAGATGGCACCCACACATCTGACCGGGCATGACCAGGGCCT**TGG**CAGGAAAAGCAG

TCACCAAGGGCGGGTGGGCAGCCCCCACCCCCACA**GGG**CAGCTGCTGGAGGACTGGCAGCCAGC

CAGCCCCGTTCCTTTTGGCTCCCTGAAG**GGG**TTTACAGATGACCTGCCTATACTTGAGTCTAGG

GTCTGTTTGCACACTTGC**CGG**CAGGACCCTCACCCAGGCTGGGTCACACTGAAGCCCAGGCCAG

AGGAAAAACACA**GGG**TTTCCACAAAGGAGCTGCCGCAATGAGGGTTTCCTTAAGGAACAGCCC**T**

GGCTCTCAAGGGTTAAAGGATAAGGCACAGCAGACAGAGGTGGGCTAGACA**AGG**ACAGATGGAA

ATTTGGTGTCTACTGGTCGCCCCAGGCAGGAATGACTCAGA**AGG**AAGCCTGGCCGTCCTGGTTC

CATG**CCA**CAGGGAAAGGCAACTGGGTCGAAATAGGCCTTGGTCTCCAGCACTATCAGTGACCCC

AGGGAGGTGACAGGC**TGG**AGCAAGTGCAGGGCAGGCAGGGGAGGGGACGCCGGCCACAGCGCAC

TCCACGGGGAA**GGG**TCTTTATGGGCCCCTCCTCGGAGAACC**CCC**GGTCTATCTGTCAGTCTGGG

ACAGGCCACCTCAACTTGCCACCGAGGACA**CCA**AAACTCTCCACAGACCCCTCTGCCCCTCTGG

GAAACCCCACTGTGCTCCAGGACACTCAAAAGGAAAGGATCCCTGGACAAGAGGTCCTGCCAGG

AACATCAGCCAAATTTTGGCCAACGACCAGCAAGGTGCACAGGGAAGAGCAGGGGCTGAAACTC

AGAGGTCCAGCATCAGCGACGCCCT**TGG**CAGCCCAGGGAACACAGGCAACG**CCT**TTTGGCTCTG

GAGTCTTAGGCTCTTCATCGGCAAACTGAGCCCAGGGGGAAGGGGCTACTACGTAGGGTTGTCA

TG**AGG**ATGAAACGAGACAGCATCTGGTGTAAAGTAGAAAAGGCATAAA**GGG**CCGGGCGCGGTGG

CTCACGCTGTAATCCCAGCACTTTTGGAGGCCCAGGC**GGG**TGGATCACCTGAGGTCAGGAGTTC

AAGA**CCA**GCTTGGCCAACCCTGTCTCCACTAAAAATAAAAAATTTTGCCGGGCGTGGTGGCGAG

CGCCTGTAATTCCAGCTACTCGGGAGGCTGAGGTAGGAGAATGGCTTGAA**CCT**GGGAGGCAGAG

GTTGCAGGGAGCCGAAATGGCAGCACTCTAGCTTGGGTGACAGAGCAAGACTCTGTCTAAAAAA

AAAAGAAAAG**CCA**TAAAGACGTGTTTGAGAAAGAGGCCTGGGAAGACGGGGGAAGGAGGGTGAT

TGAACCCGGAATGGCACTTGTGT**CGG**CCCAGGGTCATATCCCTTCATCTAAGGAT**CCT**CGTGCC

TCTAAAAAGCCACCCCGTGCTTCCTGTGGGTTTGCAAGGGCTGGCTTGGTGTATTCAGAATG**TG**
GCTTGCTGCATGAACGGACCCCGAGGGCCATGGCCCTAGAGCAGGGGCTCGCTCCAGCGGACAG
CTCTGCCTCACCGCTCCCTGCCTGTGAGTCCCGCCACGCCCTTGGTTTCTGGGCTCAGCCGTGG
AGGCAGAGGCTGG**CCT**GGCAGAGGCTGGCCTGGCAGTGCTTGACACGCAAGTGATTTGTGTCTT
CATTGCTAAGGACAAGAGGCAATGAG**AGG**ACAAGAAGTGGTTGGCCTTTTGTACGCTCAACGGG
TGGTTTTGCTACTCTGTGTCTTTTCTCTGATTTCACGGTGCTGTTAAGTGCTTAAAATATGCAC
ATCGTGTAGCTCACAGAGCCACTTCTCTGAAGGCCAGGACAGAGACCTTATAGGCTCTCTCTCC
CCCTAGTTTCAGCCTTTTACCTTAAATATACGTCTTTCTTACTGCTAGGCTGAGTTCCCG**CCC**C
AGCATGTTCTGAGAAATTGAGTCAAAATAACTGAGTCTGTTGGCACCTCATCGACGATTTCTTC
ATAGACGGTTTTTTTATTGTTGCTGTTGTTGTTGGTTTTTTGGGTTTGTTTGTTTGTTTTTTGA
GACAGAGTTTCTCTCTGTCCCCC**AGG**CTGCAGTGCAGTGGCGTGGTCTCAGCTCAGTGCAGCCT
CTGCCTCCCGGGTTCAAGAGATTCTCCTGCCTCAGCCTCCCGAGTAGCTGGGATTATAGACGCC
CAACACCACAGCGGCTAATGTTTGTATTTTTAGTAGAGATGGGGTTTCACCATGTTGGCC**AGG**C
TGGTCTCGAACTCCTGACCTCAGGTGATCCGCTCGCCTCGGCTCCCAAAGTGC**TGG**GATTATAG
GCGTGAGCTACTGTGCCTGGCCCTACTTCATAGAGGTTTAAATGCCTTTTCACCCTTTTCCTGG
AGACTCTGAAGAAGTCTCAGGAACTGGGCATTTGTGTTGCACGTG**AGG**CCTTGCAATGGCGGCC
CTGCTTGGAGGAAGGGCACTGG**CCT**GGGTTGCCCGCAGCTCCACTCCCCGTGTATGTGTTTAGG
GACCACAGAGGACAGACATCGACTCTCTGTAGAGATGCCGCCCCGCCC**AGG**TTGCAGTTTAGGT
TCCAAAAGTCCAGTGGCCAGTGGATTTTG**GGG**GAATTTGGAATAAGAAACAGCCTAGACTTTGG
AGTTGTTCATTCACTTGCAGAATTTCTACTCATGCCAGCTGCTCTGGACAGGAAGATGAATGCG
TCACAGTTCCTGCTTTTCAAAGCTCTCTAAGTTAAGTGACTTGTTTAAGATCATAGAACCCATA
AGTGAGGCAGCTGGGACTAGAACCC**AGG**TCTCCTGACTCACTGCAGCACACAGCCTTTCGGCAA
TCT**CCA**AACCAGCCCAGCCCACCGACGGAGGGAAGAACAGAAGCATTCACACA**CCC**TGCTGAGA
CAGCCATTCATTCATTCATTTGTTAATTAAACCACCATTTAGGAAACGCCTGCCTTAAGTTCCT
GACATTGTTCTAGGACACAGCACTGGATGCACACAGTGAAGAGTGAAACAGACGTGGCCCAGTC
TCT**TGG**CACTAAAATCTTGGTGCAGACAGACATCAAATAATTACGGAAATGTTCTCAACTGCAC
ATGTGGTAAATGCAGTGTGGAAAAGTACAGGGTGTGCTGAGAGCTGCATTTCGAA**TGG**CCAGAG
AGTAGGGGAGGTGCATCTGACTGACAAGTCAGGAAGGGCCCTGTG**AGG**AACCGTTCTGCGGGGA
GCTGAGGCCTGAGGCTGAGGACAGCCAGGTGGAGAAGGTGCCAGGCCTGAGCAGGCAGAGGCGG
AGCTCATGGAGAGGCAGGAAAGAGCTTGGCCCCTTGGAGGACTTGAAAGAGAAGGCAGG

gRNAs from low to high CFD

gRNA1: 1.62 w/ tandem 10 bp nearby site; tandem overlapping PAMs w/ gRNA4, everted 7 bp with gRNA7

gRNA2: 1.79 w/ tandem overlapping, PAMs 17 bp apart nearby site

gRNA3: 2.94 w/ tandem overlapping, PAMs 15 bp apart nearby site

gRNA4: 3.20 w/ tandem 9 bp nearby site; tandem overlapping PAMs w/ gRNA1, everted 8 bp w/ gRNA7

gRNA5: 3.50 w/ tandem overlapping, PAMs 4 bp apart nearby site

gRNA6: 4.13 w/ everted overlapping, PAMs 15 bp apart nearby site

gRNA7: 4.82; everted 9 bp with gRNA1, everted 8 bp w/ gRNA4

gRNA8: 5.26 w/ tandem 12 bp nearby site

gRNA9: 6.29 w/ tandem overlapping, PAMs 8 bp apart nearby site

gRNA10: 6.55 w/ everted PAMs overlapping nearby site

gRNA11: 6.83 w/ tandem overlapping, PAMs 7 bp apart nearby site; everted overlapping, PAMs 8 bp apart w/ gRNA10

gRNA12: 7.25 w/ tandem 3 bp nearby site

gRNAs 1-3, 5, 9-10, and 12 from this list selected to bind loci 1-7 in Fig. S5

Experiment 5: Process for creation of gRNAs for 8q24 and PALB2 editing and edit biosensing

8q24 risk locus (+) chr8:127,400,950-127,401,200

tctcagctccctatccataaaacagagggacgaataaactctcctcctaccacta

agaggtgtagccagagttaaTACCCTCATCGTCCTTTGAGCTCAGCAGATGAAAG

GCACTGAGAAAAGTACAAAGAATTTTTATGTGCTATTGACTTTATTTTATTTTAT

GTGGGGGAGGGAGCCGGCCCCAGCTGGAAAGCTGCTTTCTCTGAATCAAAGGGCA

GGAACCCAGCAAGTTTCTCAGGATTGGGGCC

Editing sgRNA
g259 (G->T edit): **CT**TTGAGCTCAGCAGATGAA**AGG**

sgRNAs adjacent to sgRNA used for editing
g248 (inverted overlapping): CTGAGCTCAAAGGACGATGA**GGG**
8q24gRNA1 (inverted 0 bp): GACGATGAGGGTATTAACTC**TGG** CFD 5.24
8q24gRNA2 (tandem 28 bp): actctcctccta**cca**ctaag**agg**
8q24gRNA3 (tandem 41 bp): TATTTTATTTTATGTGGGGG**AGG**

Palb2 locus (+) chr16:23,624,025-23,624,175

CCAAATTTCCCAAAGCTACA**CAC**ACG**AGATTATACACATCAGG**CACTGGAACTAT

CTGTAATAC**TGG**AACCTAAATAAAACAAAGCAGCCAAAAATTATGCTTGGTTGTT

TCATTTTTGTTTAATCCAGATTTTCCAAAATTTATCACATT

Editing sgRNA
gPalbMis1 (G->T missense): ACTGGAACTATCTGTAATAC**TGG**

sgRNAs adjacent to sgRNA used for editing
gPalbMis2 (tandem 15 bp): AAGCAGCCAAAAATTATGCT**TGG**
Palb2gRNA1 (tandem overlapping, PAMs 15 bp apart):
AGATTATACACATCAGGCAC**TGG** CFD: 23.05
Palb2gRNA2 (everted 21 bp): GTGTGTAGCTTTGGGAAATT**TGG** CFD 28.08
Palb2gRNA3 (inverted 21 bp): AAACAACCAAGCATAATTTT**TGG**
Palb2gRNA4 (tandem 21 bp): **CACACGAGATTATACACATCAGG**
